# Dual Disruption of the Immune Cytokine Spätzle Facilitates Fungal Infection of Diverse Insect Hosts

**DOI:** 10.1002/advs.202513075

**Published:** 2025-10-20

**Authors:** Shuangxiu Song, Shiqin Li, Yujuan Luo, Dongxiang Wei, Junmei Shang, Hongyun Wu, Gangqi Fang, Chengshu Wang

**Affiliations:** ^1^ Key Laboratory of Insect Developmental and Evolutionary Biology, State Key Laboratory of Plant Trait Design, CAS Center for Excellence in Molecular Plant Sciences, Shanghai Institute of Plant Physiology and Ecology Chinese Academy of Sciences Shanghai 200032 China; ^2^ School of Life Science and Technology ShanghaiTech University Shanghai 201210 China; ^3^ CAS Center for Excellence in Biotic Interactions University of Chinese Academy of Sciences Beijing 100049 China

**Keywords:** effector, immune invasion, *Metarhizium*, Spätzle, Toll pathway

## Abstract

Insect innate immunity has been well studied in *Drosophila melanogaster*. However, the mechanisms of immune invasion and host adaptation mediated by entomopathogens remain understudied. Here, it is reported that the *Drosophila* immune cytokine Spätzle (Spz, a Toll receptor ligand) can be targeted by two divergent virulence effectors (ETSs) of *Metarhizium robertsii*, a fungus that infects a wide range of invertebrates. Mechanistically, the M28‐family aminopeptidase ETS1 degrades Spz and its mature ligand form C106, while the hypothetical protein ETS6 only binds C106. Both effectors, particularly ETS6, attenuate or disable Spz interaction with its processing enzyme, the formation of the C106 dimer, and ligand‐receptor interaction. Mutagenesis of ETS6 revealed its structural uniqueness in hijacking C106. While mutant *Drosophila* lacking functional *Spz* are similarly killed by wild‐type and mutant strains of *M. robertsii*, transgenesis with either *ETS1* or *ETS6* reduced fly resistance to fungal colonization. Both effectors can target the sequence‐divergent yet structurally similar orthologous ligands of other invertebrates, unveiling a fungal mechanism for infecting and killing diverse host species. These findings reveal a rare instance of multiple effectors targeting a single immune factor in fungus‐animal interactions, and offer a mechanistic insight into the manipulation of parasite host range.

## Graphical abstract

The Toll pathway is essential for insect defense against fungal pathogens. This works reports that fungal parasites employ two divergent virulent effectors to target the Toll receptor ligand Spätzle: a metalloprotease degrading it and a hairpin‐like effector hijacking it to block signal transduction. This dual strategy enables the fungus to kill a wide range of hosts.

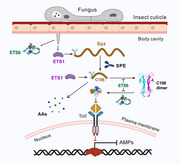

## 1 Introduction

Investigation of insect immune defenses using the fruit fly *Drosophila melanogaster* as a model has established the Toll pathway as a critical component of antifungal immunity.^[^ ^1^, ^2^ ^]^ During antifungal immune responses in flies, recognition of fungal cell wall components triggers the Spätzle (Spz) processing protease (SPE) to cleave and mature the cytokine Spzinto C106, its active Toll receptor ligand form.^[^ ^3^, ^4^ ^]^ The ligand will then form a dimer for receptor binding to activate the expression of downstream antimicrobial peptides (AMPs).^[^ ^1^, ^5^, ^6^ ^]^ Although the pathway is widely present in different insects and other invertebrates, key immune factors such as the Toll receptor and its ligand are highly divergent across insect orders and even species in terms of gene number and sequence identity.^[^ ^7^, ^8^, ^9^ ^]^ A combination of conservation and divergence also characterizes mammalian Toll‐like receptors and their ligands.^[^ ^10^ ^]^ Pathology studies have unveiled the functions of various virulence‐related genes in entomopathogenic fungi (EPF), mainly in the *Metarhizium* and *Beauveria* species.^[^ ^11^, ^12^ ^]^ For example, diverse genes have been reported in *Metarhizium robertsii* and *Beauveria bassiana* that regulate host recognition, infection structure differentiation, cuticle penetration, and/or cell wall remodeling.^[^ ^13^, ^14^, ^15^, ^16^, ^17^, ^18^ ^]^ The mechanisms by which fungal parasites cope with divergent host immune factors remain elusive.

Like plant pathogens, EPF species encode diverse effector‐like proteins in their genomes.^[^ ^19^ ^]^ Despite unresolved questions about effector mechanisms in microbe‐animal interactions,^[^ ^20^, ^21^ ^]^ accumulating evidence indicates that EPF employ effectors to suppress host immunity. For example, similar to plant pathogenic fungi,^[^ ^22^ ^]^ *B*. *bassiana* uses the LysM‐like effectors to camouflage or protect fungal cell wall chitin polymer or oligomer for immune evasion of insect hosts.^[^ ^23^ ^]^ The *M. robertsii* effectors Tge1 and Fkp1 contribute to dual suppression of pathogen recognition in *D. melanogaster* by blocking the β‐glucan receptors GNBP3/GNBP‐like 3 (GL3) and the Psh/CtsK1 protease cascade that sense pathogen‐associated danger molecules.^[^ ^24^, ^25^ ^]^ A microRNA‐like RNA in *B. bassiana* has been shown to suppress immunity by downregulating the Toll receptor ligand in mosquitoes.^[^ ^26^ ^]^ Interestingly, the *Metarhizium* spore surface protein Mcdc9 can be detected by a *Drosophila* chemosensory protein to trigger fly behavioral immune defense.^[^ ^27^ ^]^ Recent studies have unveiled that the mammalian pathogenic fungus *Candida albicans* also employs different effector‐like proteins to block host Toll‐like receptors and type I interferon signaling.^[^ ^28^, ^29^ ^]^ Overall, our understanding of gene‐for‐gene relationships in fungus‐animal interactions remains limited.

Beyond the canonical gene‐for‐gene relationship in microbe‐plant interactions, there are emerging examples of multiple pathogen effectors targeting a single host gene. For example, *Arabidopsis* RIN4, a crucial regulator of defense response, can be targeted by at least three type III effectors (AvrRpm1, AvrB, and AvrRpt2) of *Pseudomonas syringae* for phosphorylation and cleavage.^[^ ^30^ ^]^ Multiple TAL effectors (e.g., AvrX7 and pthX03) of bacterial blight pathogen *Xanthomonas oryzae* can target the promoter region of the rice susceptibility gene *OsSWEET14*.^[^ ^31^ ^]^ Likewise, two nuclear effectors (MoTHR1 and MoTHR2) were identified in the rice blast fungus *Magnaporthe oryzae* that bind to the *cis‐*elements in the promoter of *OsWRKY45* (a key regulator of salicylic acid‐mediated immunity).^[^ ^32^ ^]^ To our knowledge, this kind of instance has not been identified in fungus‐animal interactions.

As mentioned above, *Drosophila* Toll‐pathway components GNBP3, GL3, Psh and CtsK1 can be targeted by fungal effectors.^[^ ^24^, ^25^ ^]^ In this study, we asked whether the Toll ligand can be targeted by EPF to facilitate fungal infection. To answer this assumption, we used *Drosophila* Spz as bait to screen the cDNA library of *M. robertsii*. Our results show that this immune cytokine, along with highly divergent Spz homologs found in different invertebrates, can be disrupted by two fungal effectors.

## 2 Results

### 2.1 *Drosophila* Toll Receptor Ligand is Targeted by Divergent Fungal Proteins

Based on the yeast two‐hybrid (Y2H) protocol, we used Spz as bait to screen the yeast cDNA library of *M. robertsii*. This screening identified nine putative preys, termed effectors targeting Spz (ETSs; Table S1, Supporting Information). These interactions were further verified by Y2H analysis (Figure S1A, Supporting Information). Subsequent reciprocal Y2H assays with switched bait/prey roles confirmed that ETS1, ETS3 and ETS6 interact with Spz (Figure S1B, Supporting Information). Further verifications revealed that ETS3 had a self‐activation ability when co‐transformed with the empty bait vector (Figure S1C, Supporting Information). Thus, two fungal proteins remained for further experiments: the putative M28 family metalloprotease (MP) ETS1 and the hypothetical protein ETS6.

**Figure 1:**
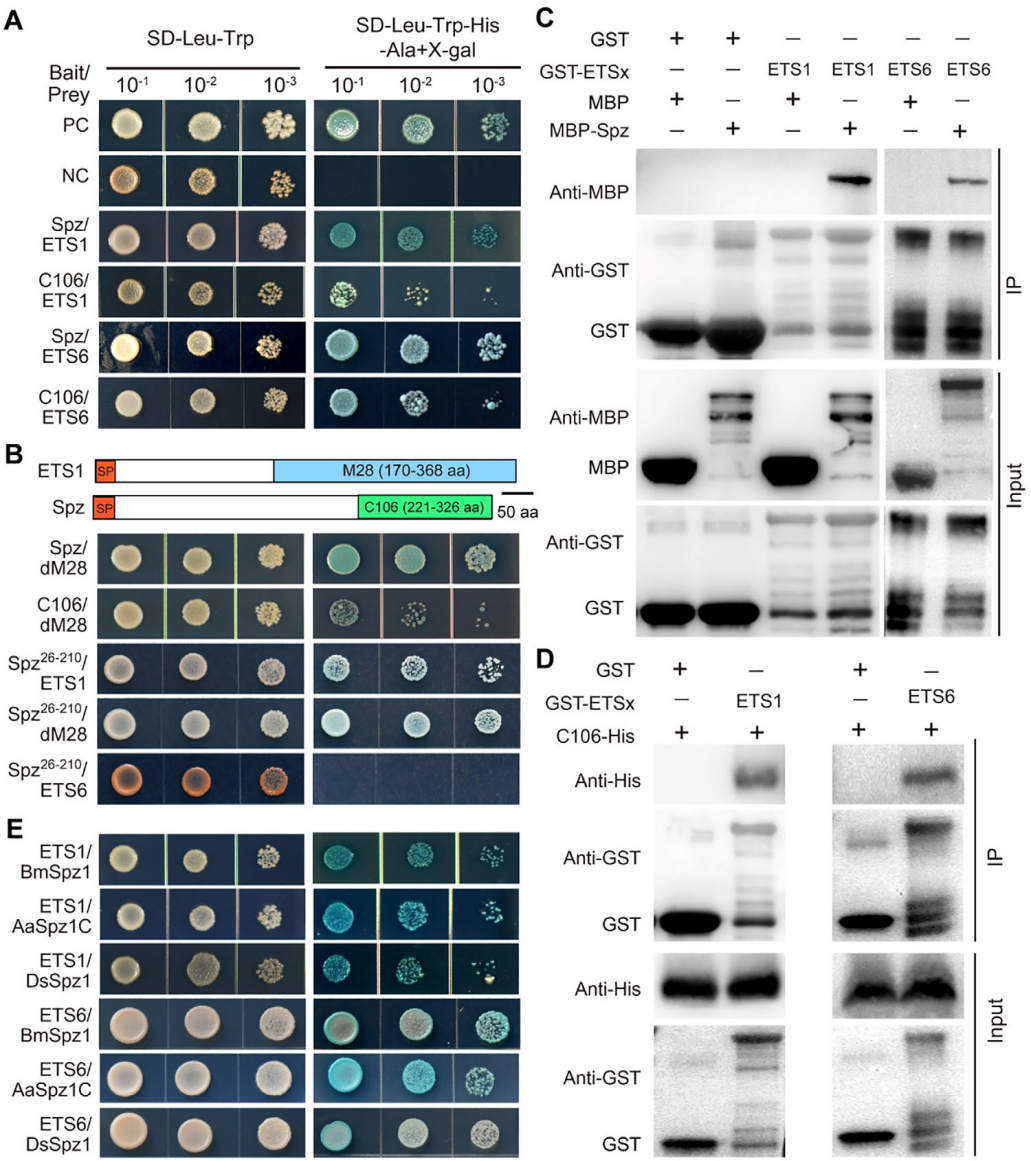
*Drosophila* Spz and its orthologs are targeted by two divergent effectors of *M. robertsii*. A) Verification of the interactions between ETS1/ETS6 and Spz/C106. The synthetic dropout (SD) medium lacking Leu and Trp was used to show the yeast cells being successfully transformed with the bait/prey plasmids whereas the SD medium lacking Leu, Trp, His and Ala plus X‐gal was used to verify the positive (cell growth) or negative (no cell growth) interaction between bait and prey proteins. B) Schematic representation and verification of the interactions between the Spz and effector domains. C,D) Protein immunoprecipitation (IP) analysis confirms the interactions between ETS1/ETS6 and Spz (C) and between ETS1/ETS6 and C106 (D). E) Y2H analysis confirms pairwise interactions between ETS1/ETS6 and the Spz homologs of BmSpz1 (*B. mori*), AaSpz1C (*A. aegypti*) and DsSpz1 (*D. suzukii*).

We found that ETS1 and ETS6 interact not only with the full‐length Spz but also with its mature form C106 (**Figure** **1**A). ETS1 contains a C‐terminus M28 domain (dM28). Further Y2H assays revealed that this dM28 region targets both Spz and C106. Both ETS1 and its dM28 region also interact with the C106 upstream region whereas ETS6 does not (Figure 1B). Yeast‐secretion assays confirmed the secretion feature of both signal‐peptide‐containing proteins (Figure S2A, Supporting Information). We next performed the heterologous expression and purification of the tag‐fused proteins for immuno‐precipitation (IP) analysis (Figure S2B, Supporting Information). The results confirmed that both ETS1 and ETS6 bind to Spz and C106 (Figure 1C,D). We also performed Co‐IP analysis using protein samples extracted from *Drosophila* after topical infection with *M. robertsii*, which revealed that glutathione S‐transferase (GST)‐tagged ETS1 and ETS6 each successfully pulled down the mature C106 ligand (Figure S1D, Supporting Information).

### 2.2 Confirming the Interactions between Spz Homologs and Fungal Effectors

Considering that the Spz‐like ligand is widely present in different insects,^[^ ^8^ ^]^ we verified that both ETS1 and ETS6 also target the Spz orthologs of other insects, including DsSpz1 of the spotted‐wing drosophila *D. suzukii*, BmSpz1 of silkworm *Bombyx mori*,^[^ ^33^ ^]^ and AaSpz1C of mosquito *Aedes aegypti* ^[^ ^34^ ^]^ (Figure 1E). Likewise, both effectors tightly interact with the maturated C‐termini of these ligand proteins (Figure S1E, Supporting Information).

Our genome survey indicated that the M28 ETS1‐like proteins are widely present in other insect pathogenic fungi such as *B. bassiana* (BBA_0 2925; termed BbETS1) and plant pathogens (Figure S3A, Supporting Information). In contrast, the ETS6‐like hypothetical genes are only present in *Metarhizium* fungi (Figure S3B, Supporting Information). We confirmed that BbETS1 also interacts with Spz and its homologs of different insects (Figure S1F, Supporting Information).

The M28 MPs comprise six subfamilies (M28A‐M28F), of which M28A, M28C‐M28F are aminopeptidases while M28B is a carboxypeptidase subfamily.^[^ ^35^ ^]^ Our survey indicated that *M. robertsii* encodes five M28 MPs, named MrM28‐1 (ETS1) to MrM28‐5 (Figure S3C, Supporting Information). We performed BLAST searches against the MEROPS database,^[^ ^35^ ^]^ and found that these five M28 MPs belong to the aminopeptidase M28A (MrM28‐4 and MrM28‐5) and M28E (MrM28‐1 to MrM28‐3) subfamilies. Phylogeny analysis demonstrated that ETS1 (i.e., MrM28‐1) is rather divergent from the other four proteins (Figure S3D, Supporting Information). Further Y2H analysis revealed that MrM28‐4 also targets both Spz and C106 while MrM28‐5 only interacts with Spz (Figure S1G, Supporting Information). We confirmed that neither ETS1 nor ETS6 directly interacts with the Toll receptor ectodomains (Figure S1H, Supporting Information).

### 2.3 Both *ETS1* and *ETS6* Play Non‐Redundant Roles in Fungal Virulence through the Suppression of *Drosophila* Antifungal Immunity

**Figure 2:**
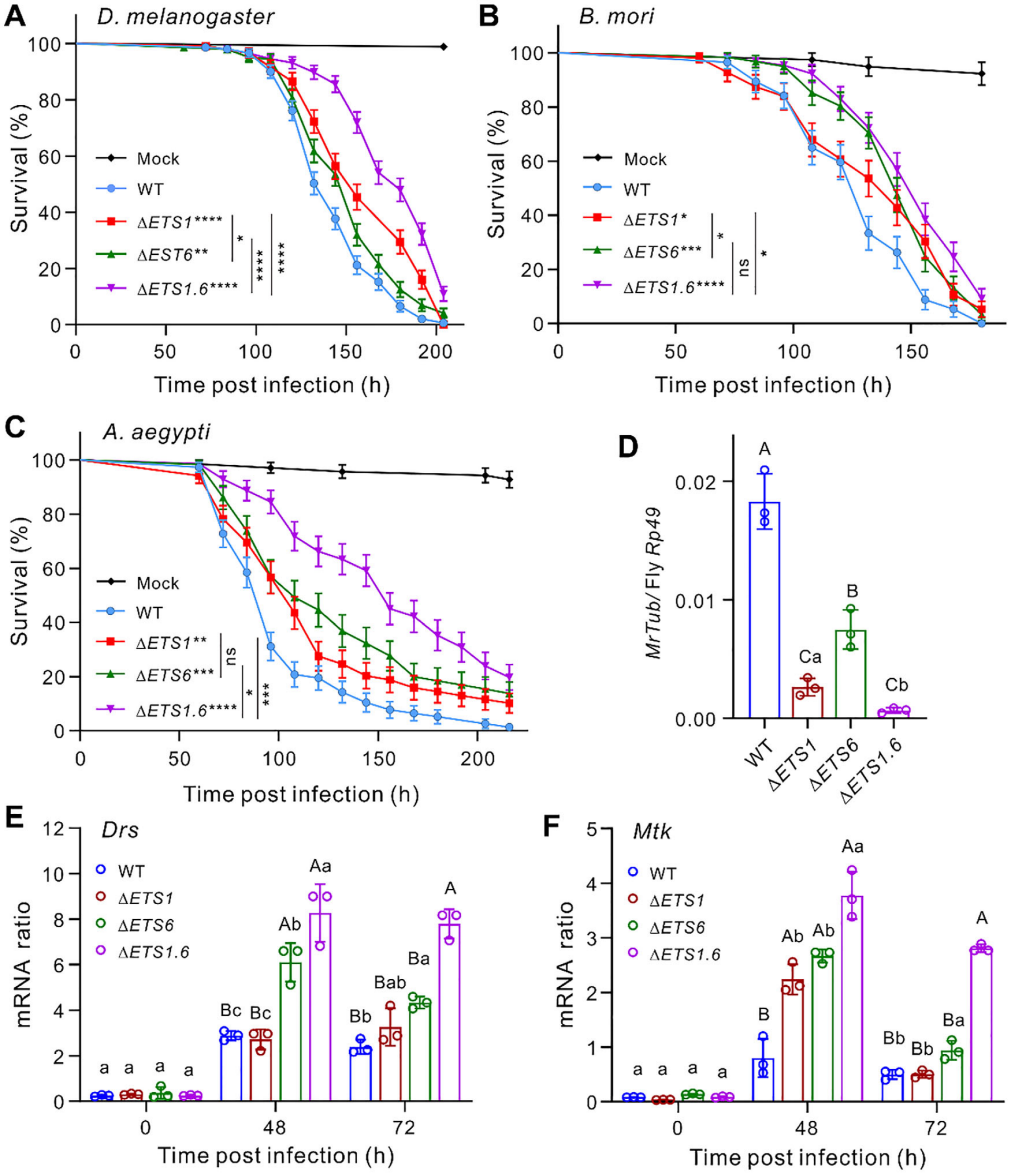
ETS1 and ETS6 are required for full virulence in *M. robertsii* by suppressing *Drosophila* antifungal immunity. A–C) Survival of *D. melanogaster* females (A), silkworm larvae (B), and *A. aegypti* females (C) after topical infections with the WT and mutant strains of *M. robertsii*. There were at least 70 insects used for each treatment. Plotted values are the mean ± SEM, obtained by Kaplan‐Meier analysis. Panels A–C: Log‐rank test: *p < 0.05; **p < 0.01; ***p < 0.001, ****p < 0.0001; ns, not significant. Asterisks labelled on strains mean the comparison of survival difference between the WT and individual mutant strains. D) Fungal load difference in the female flies after topical infections with different fungal strains for 96 h. Total DNA was extracted from the infected flies for qPCR analysis of the *M. robertsii* tubulin gene (*MrTub*) in reference to the *Rp49* gene of *D. melanogaster*. E,F) Differential expression of the antifungal *Drs* (E) and *Mtk* (F) genes in female flies over a time course following topical fungal infections. There were three independent repeats for each sample. Data are the mean ± SD. Panels D‐E: One‐way ANOVA followed by Tukey's test: different capital letters, *p <* 0.01; different lower letters, *p <* 0.05.

Our reverse transcription‐quantitative PCR (RT‐qPCR) analysis indicated that both *ETS1* and *ETS6* were transcribed by *M. robertsii* during fungal infection and artificial growth (Figure S4A,B, Supporting Information). To determine their contributions to fungal infection of insect hosts, we generated single and double deletion mutants of *ETS1* and *ETS6* in *M. robertsii* (Figure S4C, Supporting Information). The obtained mutants showed no impairment in appressorium formation (Figure S4D, Supporting Information), and had no obvious difference from the wild type (WT) or each other in artificial growth and insect cuticle penetration (Figure S4E,F, Supporting Information). We next topically infected female flies and found that the virulence of null mutants was significantly impaired (**Figure** **2**A). Compared to infection with the WT strain, survival of flies was significantly extended after topical infection with Δ*ETS1* (log‐rank test, *p <* 0.0001), Δ*ETS6* (*p* = 0.0072), and especially the double mutant Δ*ETS1.6* (*p <* 0.0001). There was no statistically significant difference in virulence between Δ*ETS1* and Δ*ETS6* whereas the Δ*ETS1.6* double mutant exhibited a more severe defect than either single mutant. Similar results were obtained during injection assays in female flies (Figure S5A, Supporting Information). Topical infections of the silkworm larvae and *A. aegypti* females also revealed that Δ*ETS1*, Δ*ETS6*, and especially Δ*ETS1.6* exhibited significantly reduced virulence against these insects compared to the WT strain (Figure 2B,C). Fungal load assays confirmed that deletion of *ETS1* and/or *ETS6* significantly impaired fungal ability (One‐way ANOVA, *p <* 0.01) in colonizing flies (Figure 2D).

We also performed deletion of *BbETS1* and overexpression (OE) of *ETS6* in *B. bassiana* (which lacks an *ETS6‐*like gene). Survival assays confirmed that *BbETS1* is required for full virulence in *B. bassiana* (*p <* 0.0001) while the potency of two independent *ETS6*‐OE mutants was significantly (*p <* 0.0001) increased against flies (Figure S5B, Supporting Information). We next examined the expression of AMPs in flies after topical fungal infections for 48 and 72 h, and found that the antifungal *Drosomycin* (*Drs*) and *Metchnikowin* (*Mtk*) genes were significantly upregulated in flies infected with mutant strains, especially with Δ*ETS1.6* (*p <* 0.01) (Figure 2E,F). Likewise, the antifungal Baramicin gene *BaraA* ^[^ ^36^ ^]^ and Bomanin gene *BomS1* ^[^ ^37^ ^]^ were upregulated in flies infected with *Metarhizium* mutants (Figure S5C,D, Supporting Information). These data revealed therefore that both ETS1 and ETS6 are required for *Metarhizium* to suppress fly antifungal immune responses.

### 2.4 Fungal Effectors Degrade Spz or Interfere its Interaction with Associated Proteins

**Figure 3:**
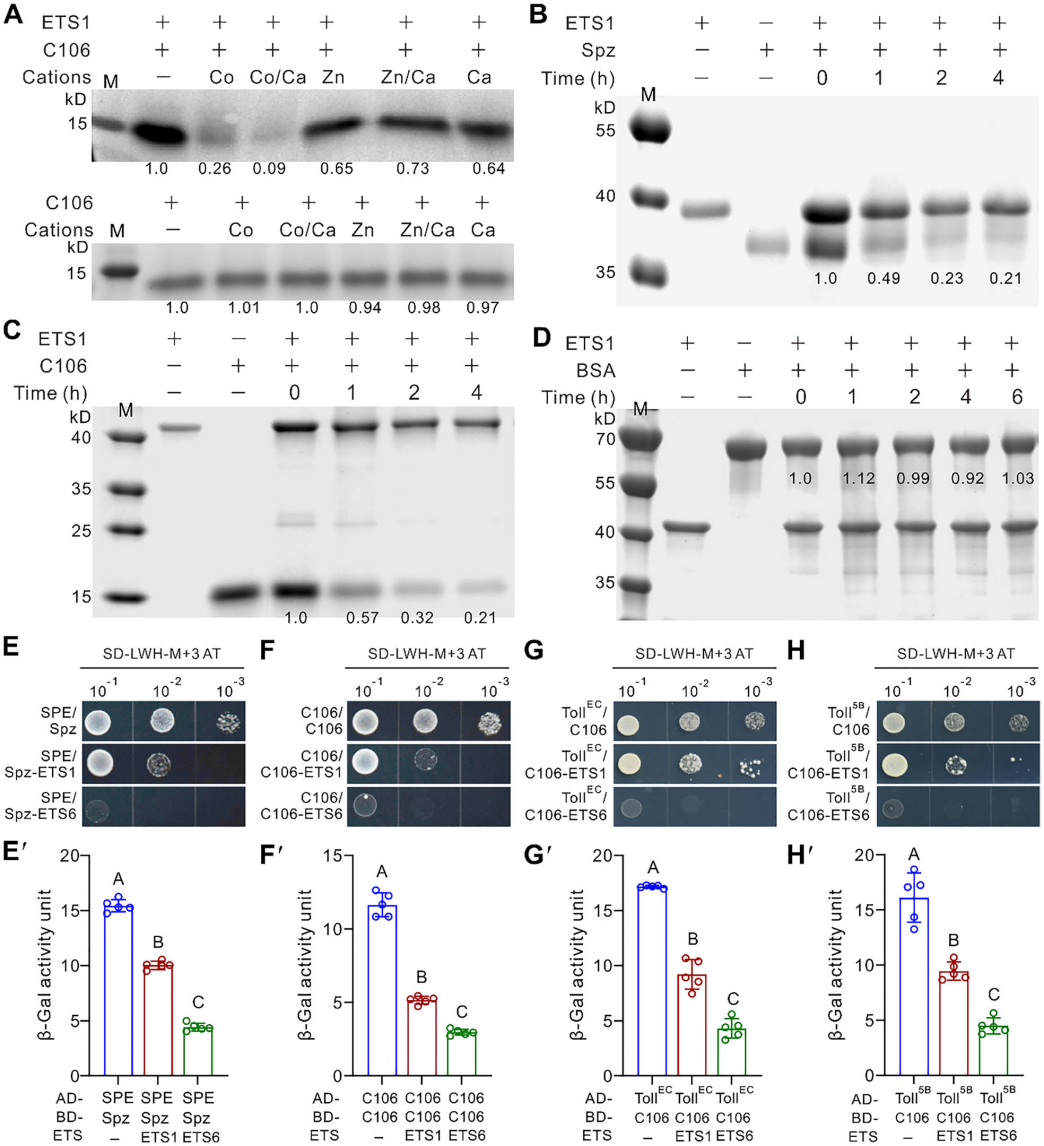
Ligand degradation and blockage of immune protein interactions by fungal effectors. A) Verification of the effects of cation cofactors on the activity of ETS1 in degrading C106 over a 5 h period. Each reaction system (50 µl) contained the final concentration of 1 µg µL^−1^ ETS1, 2 µg µL^−1^ C106, and 1 mM of each cation. The control treatments were included without the addition of ETS1 (lower panel). B,C) Degradation of Spz (B) and C106 (C) by ETS1 over a time course. The reaction system (50 µl) contained the final concentration of 2 µg µL^−1^ ETS1, 2 µg µL^−1^ Spz or C106, and 1 mM each of CoCl_2_ and CaCl_2_. D) No degradation of BSA by ETS1. The reaction system (50 µl) contained the final concentration of 2 µg µL^−1^ of ETS1, 5 µg µL^−1^ BSA and 1 mM CoCl_2_ and CaCl_2_. Individual proteins, ETS1, Spz, C106 and BSA, were added to the reaction buffer simultaneously and incubated till the reactions were complete before being loaded for protein gel analysis. Band intensity was quantified using ImageJ. E‐H) Y3H assays showing the inhibition of ETS1/ETS6 on the interactions between SPE and Spz (E), between C106 itself (F), between C106 and Toll ectodomain Toll^EC^ (28‐397 aa) (G), and between C106 and Toll ectodomain Toll^SB^ (28‐668 aa) (H). The upper panels show the yeast cell survivals (E‐H) and the lower panels (E′‐H′) show the corresponding β‐galactosidase activity. ETS1/ETS6 inhibition activity was activated by growing yeast cells on/in the SD‐Leu‐Trp‐His‐Met medium plus 1 mM 3‐AT. There were five independent repeats for each sample. Data are the mean ± SD. Different capital letters labeled above columns show the results (*p <* 0.01) of the one‐way ANOVA followed by Tukey's test.

Having indicated above that ETS1 is a putative aminopeptidase, we next performed the proteolytic analysis of Spz and C106 using the purified ETS1 enzyme. After optimizing the cation cofactors as Co^2+^/Ca^2+^ using purified proteins (**Figure** **3**A), we found that ETS1 degraded both Spz and C106, reducing their levels by approximately half within 1 h and by 80% after 4 h (Figure 3B,C). In contrast, ETS1 did not degrade bovine serum albumin (BSA) (Figure 3D), demonstrating its specificity for Spz/C106.

We next conducted the yeast‐three hybrid (Y3H) assays to determine whether ETS1/ETS6 influences the interaction between Spz/C106 and its binding partners by examining the yeast cell viability and β‐galactosidase activity.^[^ ^25^ ^]^ As a result, ETS1, and especially ETS6, significantly (*p <* 0.01) inhibited the interaction between the processing enzyme SPE and its substrate Spz (Figure 3E). In addition, the formation of C106 dimer (Figure 3F), and the binding of C106 to the Toll receptor ectodomains were substantially hindered by ETS1, and especially by ETS6 (Figure 3G,H). Unsurprisingly, SPE does not target C106 and shows no self‐interaction (Figure S1I, Supporting Information).

### 2.5 Structure Prediction and Verification of Effector Functional Conservation

**Figure 4:**
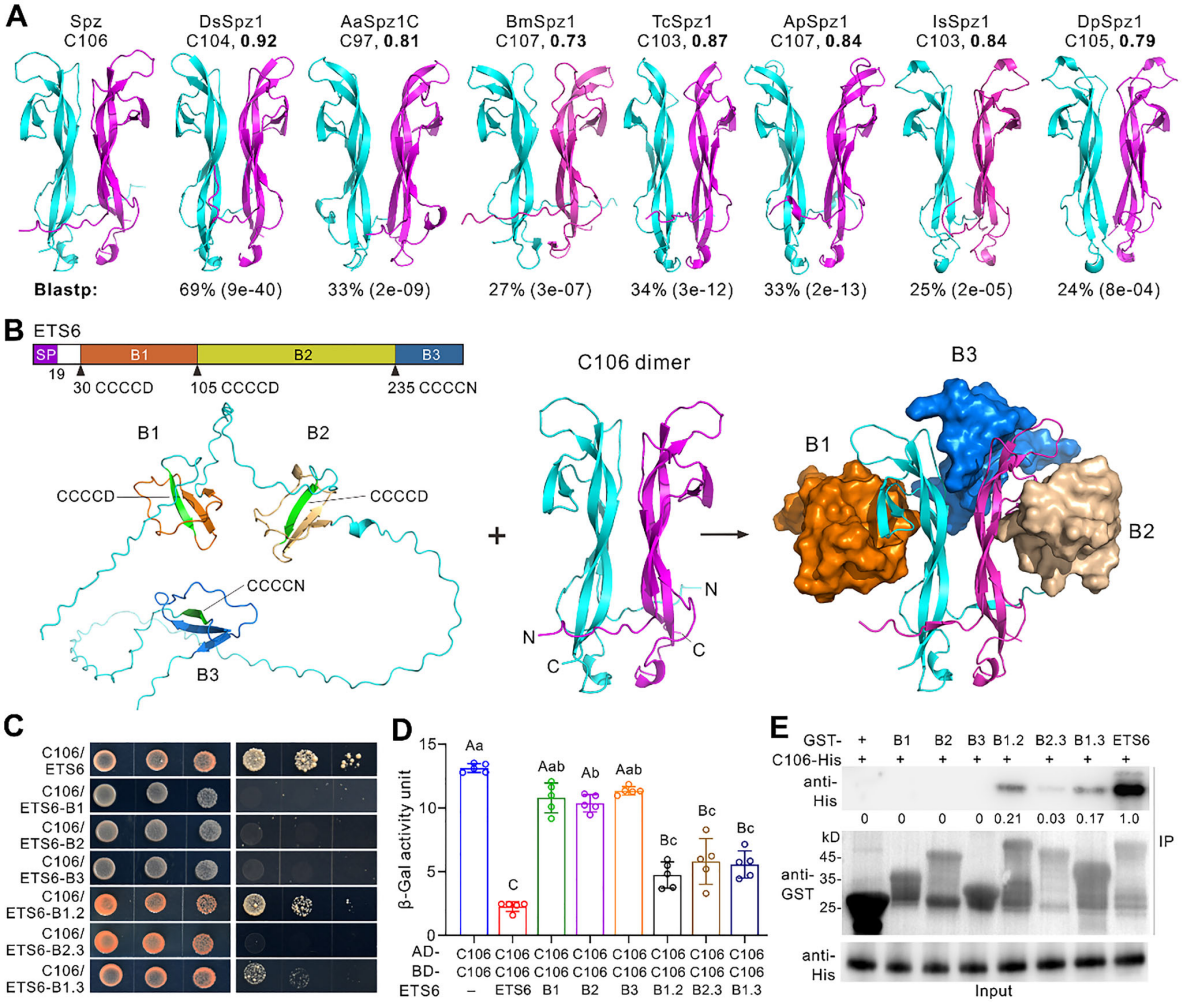
Protein structure modeling and functional verification. A) Dimer structures predicted for the Toll ligands of different invertebrates. Different ligands are DsSpz1‐C104 from the spotted‐wing drosophila *D. suzukii*; BmSpz1‐C107 from the silkworm *B. mori*; TcSpz1‐C103 from the red flour beetle *Tribolium castaneum*; ApSpz1‐C107 from the pea aphid *Acyrthosiphon pisum*; IsSpz1‐C103 from the deer tick *Ixodes scapularis* and DpSpz1‐C105 from the water flea *Daphnia pulex*. C‐terminal amino acid lengths are shown as reported or deduced, and the TM‐scores are shown in bold in reference to the C106‐dimer structure of *D. melanogaster*. The values below each structure show primary sequence identify (e‐values in parenthesis) compared to the *D. melanogaster* C106. B) Structure modeling and prediction of ETS6, C106 dimer, and the complex of ETS6/C106. Three CCCCD/N motifs of ETS6 are indicated in forming the start β‐sheet (labeled in green) of three blocks (B1–B3). SP, signal peptide. C) Y2H assays show the impaired interactions between C106 and truncated ETS6 isoforms. Different isoform blocks of ETS6 are as shown in panel (B). D) Y3H assays of β‐galactosidase activity show that the truncated ETS6 isoforms are impaired in inhibiting C106 dimerization. There were five independent repeats for each sample. Data are the mean ± SD. Different capital (*p <* 0.01) or lower (*p <* 0.05) letters labeled above columns show the results of the one‐way ANOVA followed by Tukey's test. E) Co‐IP assay shows that the truncated ETS6 isoforms are impaired in interaction with C106. Different truncated isoforms of ETS6 were expressed in fusion with a GST tag for immuno‐precipitation (IP) analysis.

Further Y2H analysis indicated that, like C106, the C‐termini of DsSpz1 (C104), AaSpz1C (C97), and BmSpz1 (C107) each self‐interact (Figure S6A, Supporting Information), indicating their similar abilities to form dimers. We next selected seven Toll receptor ligands that have been reported elsewhere for sequence alignment and structure predictions. Intriguingly, the primary sequences of these ligands are highly divergent from other each (Figure S6B, Supporting Information). However, the predicted dimer structures [with the pLDDT (predicted local distance difference test) values being largely more than 90] were highly similar to that of C106 (**Figure** **4**A). The estimated template modeling (TM) scores were all above 0.7, indicating a high degree of structural similarity.^[^ ^38^ ^]^ Unsurprisingly, the predicted dimer structure of *D. suzukii*’s DsSpz1‐C104 is mostly similar to that of C106 (TM‐score = 0.92). However, those of the non‐insect water flea *Daphnia pulex* (DpSpz1; 0.79) and tick *Ixodes scapularis* (IsSpz1; 0.84) are also well aligned to the C106 dimer structure. BLASTp analyses confirmed a high level of sequence divergence between these proteins and *Drosophila* C106 (Figure 4A). For example, DpSpz1 only has an amino acid identity of 24% (e‐value, 0.003) to Spz, and IsSpz1 has a 26% identity (3e‐08) to Spz.

We then predicted the structure of the C106 dimer in complex with ETS6 and found that ETS6 putatively forms a structure with three blocks, each containing three β‐sheets starting from the CCCCD/N motifs that may hijack the C106 dimer (Figure 4B; Figure S6C, Supporting Information). To verify its predicted structure and function, we obtained truncated ETS6 isoforms. The subsequent Y2H analysis revealed that no single block of ETS6 could interact with C106, whereas the double‐block isoforms 1 and 2 (B1.2) and fused B1.3 but not B2.3 weakly bound C106 (Figure 4C). Two domain forms of ETS6 were predicted to still form hairpin‐like structures (Figure S6D, Supporting Information). Further Y3H β‐galactosidase activity assays confirmed that single‐block ETS6 isoforms could not inhibit C106 dimerization, whereas two‐domain isoforms significantly (*p <* 0.01) impaired the formation of C106 dimer (Figure 4D). We expressed truncated ETS6 proteins (Figure S7A, Supporting Information), and the following Co‐IP analysis verified that ETS6 truncations disabled or substantially reduced its ability to interact with C106 (Figure 4E). To verify the functional conservation of ETS1, we expressed DsSpz1‐C104, AaSpz1C‐97, and BmSpz1‐C107 for proteolytic degradation (Figure S7B, Supporting Information). The results indicated that ETS1 effectively degraded these C106‐like ligands derived from different insects (Figure S7C–E, Supporting Information).

### 2.6 Confirming of *ETS1* and *ETS6* Functions Using *Drosophila* Mutants

**Figure 5:**
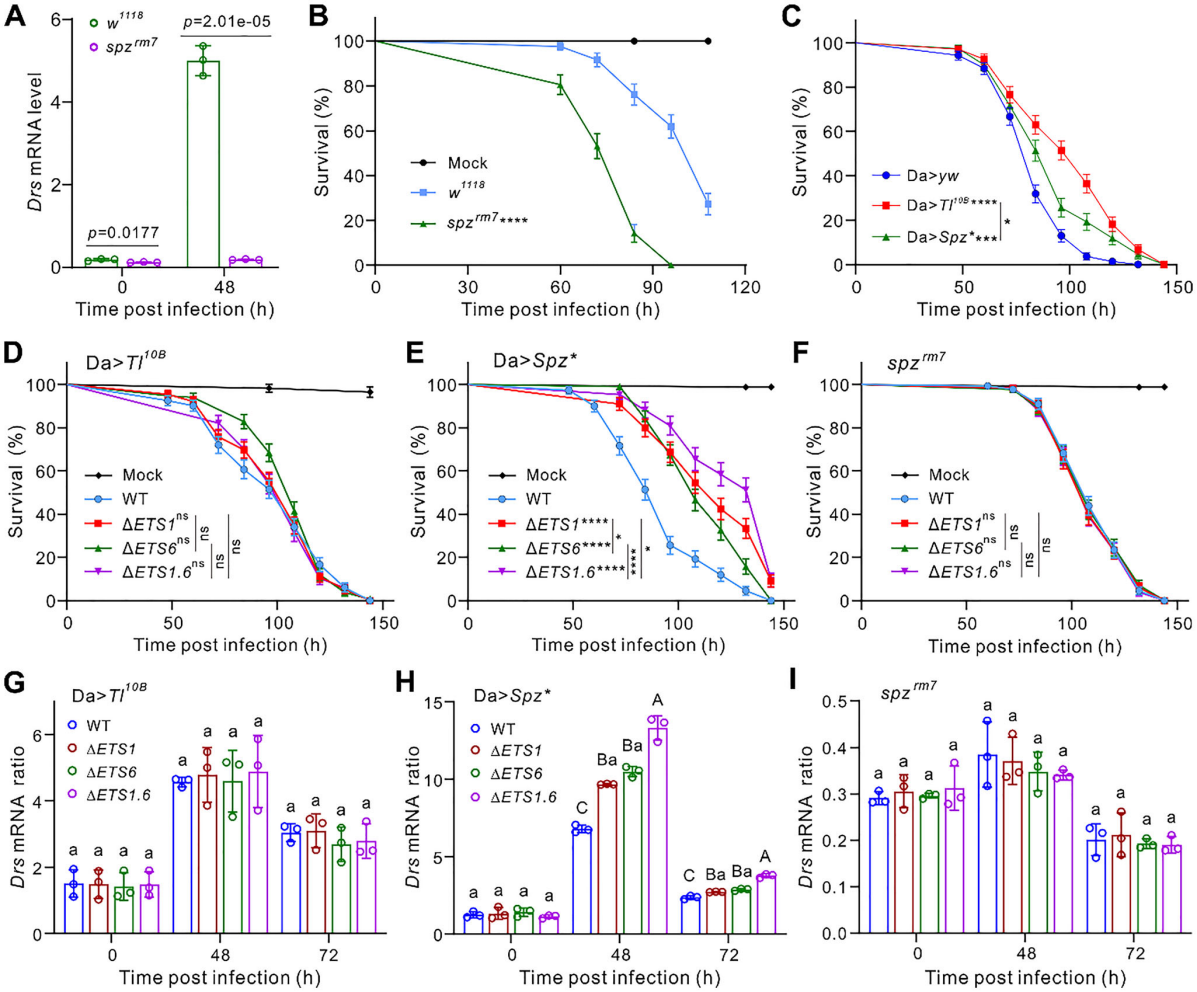
Confirmation of effector functions through the use of *Drosophila* mutants for survival and AMP gene expression assays. A) Expression of *Drs* was abolished in the *Spz‐*deficient flies upon fungal infection. There were three independent repeats for each sample. Data are the mean ± SD. *p* values labeled above are estimated by two‐tailed Student's *t‐*test. B) Constitutively activating *Toll* (Da>*Tl^10B^*) or *Spz* (Da>Spz*) in *Drosophila* increased female fly antifungal abilities against the WT of *M. robertsii*. D–F) Survival of Da>*Tl^10B^* (D), *Da*>Spz* (E) and *spz^rm7^* (F) female flies after topical infections with the WT and mutant strains of *M. robertsii*. G–I) Differential expression of *Drs* in Da>*Tl^10B^* (G), Da>Spz* (H) and *spz^rm7^* (I) female flies after topical infections with WT and mutant strains of *M. robertsii* for different times. Panels B‐F: There were more than 70 flies used for each treatment. Plotted values are the mean ± SEM. Log‐rank test: *p < 0.05; ***p < 0.001; ****p < 0.0001. ns, not significant. Panels G‐I: There were three independent repeats for each sample. Data are the mean ± SD. One‐way ANOVA followed by Tukey's test was conducted between samples examined at the same time: different capital letters, *p <* 0.01; different lower letters, *p <* 0.05.

Having shown above that both ETS1 and ETS6 are involved in dual inactivation of Spz, we subsequently assessed fungal virulence against *Spz* and *Toll* mutant flies. Consistent with previous analyses,^[^ ^2^, ^4^, ^39^ ^]^ *Spz* null flies (s*pz^rm7^*) only exhibited the basal expression of *Drs* after fungal challenge (**Figure** **5**A), and succumbed much faster than *w^1118^* to infection with the WT strain of *M. robertsii* (Figure 5B). In contrast, overexpressing *Spz* (Da>Spz^*^) and *Toll* (Da>*Tl^10B^*) in *Drosophila* significantly increased fly antifungal abilities (*p <* 0.001) (Figure 5C), which is in line with previous findings.^[^ ^40^, ^41^ ^]^ Interestingly, Da>*Tl^10B^* females were equally susceptible to topical infection with the WT, Δ*ETS1*, Δ*ETS6* and Δ*ETS1.6* strains of *M. robertsii* (Figure 5D). Topical infection of Da>Spz^*^ females resulted in survival patterns being similar to those of *w^1118^* flies after challenge with the WT and mutant strains. For example, Δ*ETS1.6* exhibited the lowest infectivity in Da>Spz^*^ flies (*p <* 0.0001) compared to the WT strain (Figure 5E). In contrast, s*pz^rm7^* lacking a functional ligand succumbed uniformly to both WT and mutant strains (Figure 5F). The detection of *Drs* expression largely supported these survival data, as its transcription was similarly activated in Da>*Tl^10B^* and *spz^rm7^* flies, but was significantly upregulated in Da>Spz^*^ flies after topical infection with mutant strains compared to the WT of *M. robertsii* (Figure 5G–I). Da>Spz^*^ flies infected with Δ*ETS1.6* exhibited the highest level of *Drs* expression, reaffirming the functional importance of both ETS1 and ETS6 in inactivating Spz and, consequently, suppressing AMP expression.

### 2.7 Overexpressing ETS1 and ETS6 in *Drosophila* Subverts Antifungal Immunity

**Figure 6:**
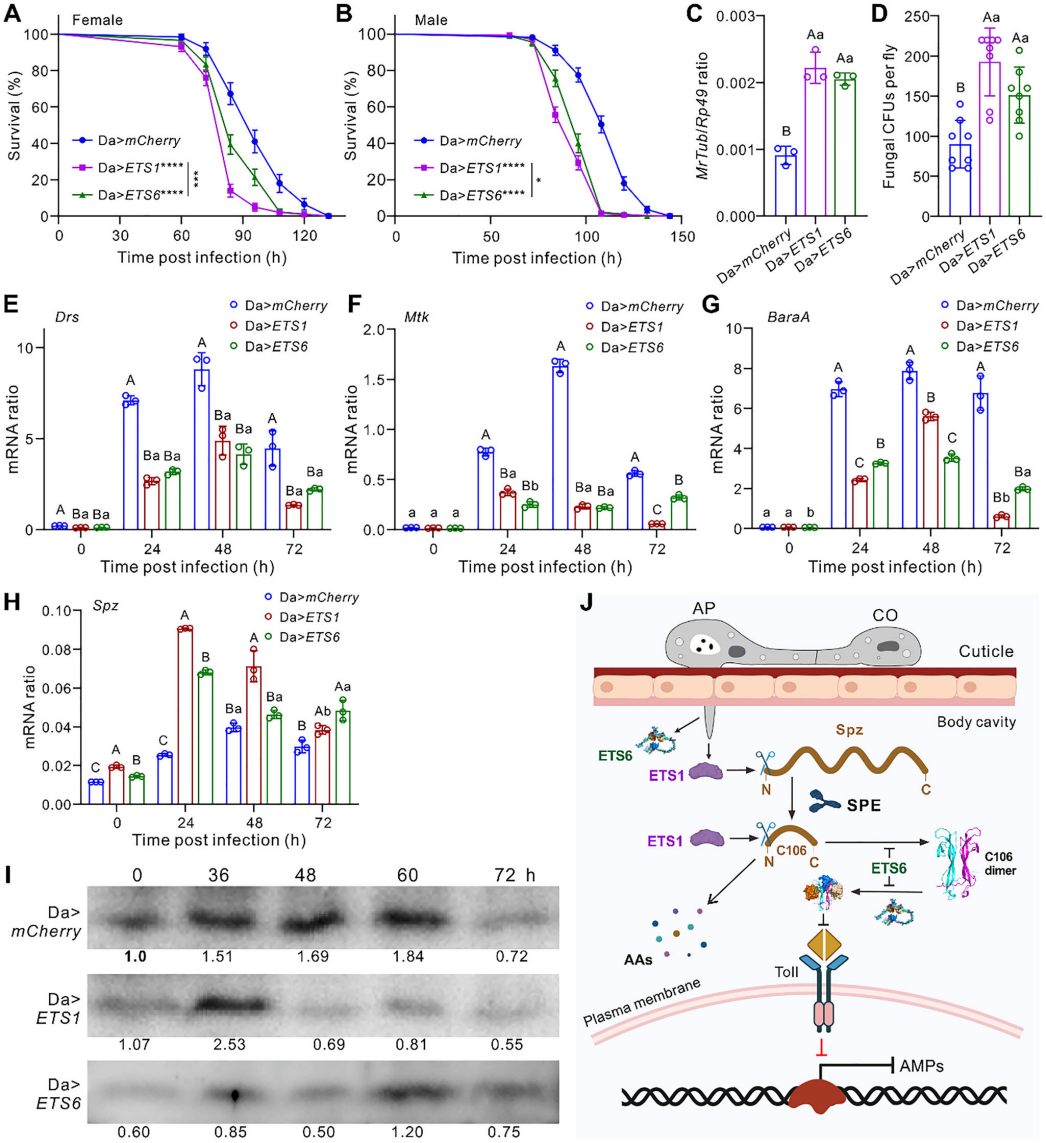
Transgenic expression of fungal effectors in *Drosophila* inhibits antifungal immunity. A,B) Differential survivals of the *ETS1‐* and *ETS6‐*transgenic female (A) and male (B) flies after topical infection with the WT strain of *M. robertsii*. Da>*mCherry* flies were included as a mock control. There are more than 70 flies used for each treatment. Plotted data are the mean ± SEM. Log‐rank test: *p < 0.05; ***p < 0.001; ****p < 0.0001. C,D) Transgenic expression of *ETS1* and *ETS6* in *Drosophila* females impaired their ability in resisting fungal colonization as assessed by qPCR analysis (C) and fungal CFU counting (D). Flies were topically infected for 72 h. E–G) Downregulation of *Drs* (E), *Mtk* (F), and *BaraA1* (G) in the *ETS1*‐ and *ETS6‐*transgenic female flies after topical fungal infection for different times. H) Upregulation of *Spz* in the *ETS1‐* and *ETS6‐*transgenic female flies after topical fungal infection for different times. I) Differential accumulation of C106 in the *ETS1‐* and *ETS6‐*transgenic flies after topical fungal infection for different times. The hemolymph samples were collected from flies (50 males plus 50 females) at each time point and used for the blotting analysis using the anti‐C106 antibody. J) Schematic of the dual strategy mediated by ETS1 and ETS6 in suppressing *Drosophila* antifungal immunity. AP, appressorium; CO, conidium; AAs, amino acids. Panels C‐H: There were three independent repeats for each sample. Data are the mean ± SD. Different capital (*p <* 0.01) or lower (*p <* 0.05) letters labeled above columns show the results of the one‐way ANOVA followed by Tukey's test.

Finally, we generated transgenic *Drosophila* lines, and survival assays revealed that both *ETS1*‐ and *ETS6*‐overexpressing females and males succumbed significantly faster (*p <* 0.0001) than the control flies to topical challenge with the WT *M. robertsii* (**Figure** **6**A,B). Consistently, fungal load assays revealed that overexpressing either *ETS1* or *ETS6* in *Drosophila* significantly (*p <* 0.01) impaired the host's ability to resist fungal colonization (Figure 6C,D).

We examined the expression of several AMP genes and found significant downregulation of the antifungal genes *Drs*, *Mtk*, *BaraA*, *Bomanin*, and *Daisho* ^[^ ^42^ ^]^ in both *ETS1‐* and *ETS6‐*transgenic flies following fungal infection (Figure 6E–G; Figure S8A–D, Supporting Information). Interestingly, *Spz* was upregulated in *ETS1*‐ and *ETS6*‐overexpressing flies (Figure 6H), implying a compensatory immune response in these flies. The inductive expression of *Spz* is independent of Toll pathway components, such as ModSP, Psh, Toll, and MyD88 (Figure S8E–G, Supporting Information). Fly hemolymph was collected for Western blot analysis (Figure S7F, Supporting Information), and the results revealed that C106 turnover was rapidly reduced in *Da*>*ETS1* flies but not *Da*>*ETS6* flies (Figure 6I), supporting the proteolytic activity of ETS1. Unsurprisingly, injection of female flies with the Gram‐negative bacterial cells of *Erwinia carotovora carotovora 15* (*Ecc15*) did not result in significant survival differences between control and transgenic flies (Figure S8H, Supporting Information). The expression of *Diptericin*, a downstream effector of the Imd (immune deficiency) pathway, did not differ significantly after injection with *Ecc15* (Figure S8I, Supporting Information). However, septic injury of *Drosophila* females with the Gram‐positive bacterium *Micrococcus luteus* resulted in survival patterns similar to those from *M. robertsii* topical infection. Thus, flies overexpressing *ETS1* (*p* < 0.0001) or *ETS6* (*p* < 0.001) were more susceptible than the mock control (Figure S8J, Supporting Information). Consistently, *Drs* expression was significantly reduced in transgenic flies after injection with *M. luteus* cells (Figure S8K, Supporting Information).

## 3 Discussion

Twenty years after the discovery of Spz cytokine's role in Toll‐mediated antifungal responses,^[^ ^2^, ^4^ ^]^ we uncovered in this study that *Metarhizium* pathogens have evolved a dual strategy to disrupt Spz with two divergent effector proteins. To our knowledge, this is the first instance of two genes acting against one in fungus‐animal interactions. The M28E subfamily aminopeptidase ETS1 degrades Spz and its mature form C106 whereas ETS6 hijacks the C106 dimer, if C106 is leaked, to disable its function as a Toll receptor ligand (Figure 6J). Unsurprisingly, both ETS1 and ETS6 are non‐redundantly required for fungal virulence. Deletion of fungal effector genes in *Metarhizium* or overexpression of *ETS1*/*ETS6* in flies led to the substantial up‐ or down‐regulation of AMP genes in *Drosophila* after fungal challenges. Intriguingly, early‐diverged *Beauveria* species only contain an *ETS1* ortholog while exogenous expression of *ETS6* in *B. bassiana* enhanced fungal virulence. Our data also reveal that both ETS1 and ETS6 can target divergent C106‐like ligands from other invertebrates, which paints an insightful picture to help understand the evolution of fungal host range.

Metalloproteases have been characterized by their virulence attributes in diverse plant and animal pathogens.^[^ ^43^, ^44^ ^]^ For example, plant defensive chitinases can be cleaved by the M36 family fungalysin encoded by fungal pathogens.^[^ ^45^, ^46^ ^]^ A M35 family MP secreted by *M. robertsii* degrades insect immune prophenoloxidases into non‐functional fragments.^[^ ^47^ ^]^ Human pathogenic fungi such as the dermatophytes secrete the M28 MP to decompose keratin.^[^ ^48^ ^]^ We found that M28E subfamily members ETS1 and BbETS1 interact with both Spz and its homologs of different insects, as well as with their mature ligand forms. Functional characterization of the mold fungus *Aspergillus oryzae* M28 LapA revealed that its N‐terminal domain governs substrate sorting and requires zinc for activity.^[^ ^49^ ^]^ We found that the combinational use of CoCl_2_ and CaCl_2_ resulted in the most efficient degradation of C106 by ETS1, followed by the use of cations Zn^2+^, Co^2+^, and Ca^2+^. Future biochemical investigations are still needed to determine the substrate specificity control of ETS1/BbETS1. The putative M28A subfamily MrM28‐4 of *M. robertsii* also targets both Spz and C106, implying its additive contribution to inactivating this ligand; however, which requires further investigation.

In contrast to the broad distribution of *ETS1*‐like genes across fungi, *ETS6* homologs appear to be restricted to *Metarhizium* fungi. In support of virulence decline after *ETS6* deletion in *M. robertsii*, exogenous *ETS6* expression significantly enhanced the virulence of *B. bassiana* against flies. ETS6 specifically targets the C106 region of Spz but not its upstream region, implying that ETS6 functions downstream of SPE in the *Drosophila* Toll pathway. C106 can also be degraded by ETS1, suggesting a certain degree of substrate competition between ETS1 and ETS6. Nevertheless, the emergence of the *ETS6* gene likely confers a synergistic effect in *Metarhizium* fungi by enabling them to hijack the ligand. Therefore, it is not surprising that the Da>Spz* flies exhibited a similar mortality pattern to *w^1118^* flies whereas the *spz^rm7^* allele showed equal susceptibility after topical infections with the WT and mutant strains. Intriguingly, given that the receptor‐overexpression flies (Da>*Tl^10B^*) had a survival advantage when challenged with the WT *Metarhizium*, their survival rates did not differ significantly when infected with WT versus *ETS1/ETS6* mutant strains. In support, similar induction of *Drs* expression was evident in Da>*Tl^10B^* flies. Overexpressing Toll‐like receptors (TLRs), particularly *Tlr4*, in mice enhanced bacterial clearance but concurrently induced detrimental host effects such as immune disorders and oxidative stress.^[^ ^50^ ^]^ In this respect, Da>*Tl^10B^* flies may also exhibit immune dysfunction, leading to their similar survival rates upon fungal infections. Nevertheless, the underlying mechanism requires further investigation.

Similar to the enigmatic emergence of lineage‐specific virulence effectors in plant pathogens,^[^ ^51^ ^]^ the evolutionary origin and functional acquisition of *ETS6* in *Metarhizium* remains unresolved. Given that *Metarhizium* lineage diverged after the speciation of *Beauveria* species,^[^ ^11^ ^]^ *ETS6* likely emerged in a *Metarhizium* ancestor. It is noteworthy that the acridid‐specific pathogen *M. acridum* also possesses both ETS1 and ETS6 orthologs, indicating that these effectors do not determine fungal host specificity. The inability of *M. acridum* to form appressoria on non‐acridid insects indicates that this fungus cannot enter the body cavity of those non‐hosts.^[^ ^52^, ^53^ ^]^ Thus, these effectors cannot function within non‐host body cavities.

Unlike the basal expression of *Drs* in the *spz^rm7^* mutant *Drosophila*,^[^ ^4^, ^39^ ^]^ we found that antifungal gene expressions were significantly reduced but not completely abolished in *ETS1‐* or *ETS6*‐overexpressing flies after fungal challenge. In support, *Spz* was considerably upregulated in both *ETS1‐* and *ETS6*‐transgenic flies, and a reduced level but not a complete loss of C106 ligand was detected in the hemolymph of Da>*ETS1* flies. In Da>*ETS6* flies, however, C106 upregulation was evident starting from 48 h after *Metarhizium* infection. ETS6 substantially suppresses C106 dimerization or hijacks the C106 dimer, thereby preventing its binding to the Toll receptor. Thus, the upregulated C106 in Da>*ETS6* flies would be largely non‐functional. Overall, the data suggest a compensatory mechanism of immune responses in *Drosophila*. Likewise, disruption of the *GNBP3* gene resulted in the upregulation of *Psh* in *Drosophila* to compensate for impaired activation of the antifungal Toll pathway.^[^ ^24^, ^39^ ^]^ Taken together, our data suggest that the thresholds of circulating immune factors are tightly monitored and finely tuned in *Drosophila*; however, the mechanisms governing these hemostatic responses require further investigations.

Despite the conservation of innate immune pathways in animals,^[^ ^54^ ^]^ immune defense genes are among the fastest evolving members in many species, particularly the downstream AMP genes in insects.^[^ ^55^ ^]^ Thus, the number and presence/absence of immune gene families vary considerably in diverse invertebrates. For example, *D. melanogaster* has nine Toll receptors and six Spz paralogs whereas the water flea *Daphnia pulex* has five Toll and four Spz members.^[^ ^7^ ^]^ Notably, *Drosophila* Spz5 is also a ligand of Toll.^[^ ^56^ ^]^ However, its function in antifungal immunity is still elusive. It remains to be determined whether ETS1/6 also target Spz5 or other paralogs. Most strikingly, despite the highly divergent ligand sequences between and even within different orders of insects or non‐insect invertebrates, these ligands form highly similar structures that can be targeted by ETS6. Likewise, emerging evidence has shown that even proteins sharing no primary sequence similarity can form highly similar structures,^[^ ^57^, ^58^ ^]^ suggesting a convergent evolution of functional folds, which can be targeted by individual parasite effectors.

In conclusion, we demonstrate that *Metarhizium* fungi have evolved two distinct effectors to sabotage the *Drosophila* antifungal Toll‐receptor ligand via proteolytic degradation and molecular hijacking. The results of this study not only advance our understanding of fungus‐animal interactions but also shed light on the mechanistic control of parasite host range against diverse hosts.

## 4 Experimental Section

### Microbial Strains and Culturing

The WT strain ARSEF 2575 and mutants of *M. robertsii*,^[^ ^59^ ^]^ and WT strain ARSEF 2860 and mutants of *B. bassiana* were grown on potato dextrose agar (PDA; BD Difco) or a complete medium^[^ ^60^ ^]^ for sporulation at 25 °C for two weeks. The WT strain was grown in Sabouraud dextrose broth (SDB; BD Difco) for RNA extraction after incubation for three days at 200 rpm and 25 °C. The sterile cicada wings were placed on the minimum medium (MM; 6 g L^−1^ NaNO_3_, 0.52 g L^−1^ KCl, 0.52 g L^−1^ MgSO_4_⋅7H_2_O, 0.25 g L^−1^ KH_2_PO_4_) agar for inoculation of the WT and mutant strain to examine fungal penetration ability.^[^ ^16^ ^]^

The budding yeast *Saccharomyces cerevisiae* strains Y2H Gold (Weidi Biotech) and AH109 (TaKaRa Bio) were maintained on the synthetic dropout (SD) media (Sigma‐Aldrich) and used for Y2H and Y3H assays, respectively. Different *Escherichia coli* strains were cultured on the Luria‐Bertani (LB) agar or in LB broth at 37 °C for gene cloning (Top10, Weidi Biotech) or protein expressions [BL21(DE3)] at 16 °C. The strain AGL1 of *Agrobacterium tumefaciens* was used for fungal transformation.^[^ ^27^ ^]^

### Insect Rearing

The *D. melanogaster* stocks *w^1118^*, *Tl*, *spz^rm7^*, UAS‐*Tl^10B^*,^[^ ^40^ ^]^ UAS‐Spz^*^,^[^ ^41^ ^]^ UAS‐*EST1*, UAS‐*ETS6*, UAS‐*mCherry* and tool lines (*w*;*Da‐Gal4;tub‐Gal80^ts^* and *w;Ubi‐Gal4;Tub‐Gal80^ts^*) were maintained at 25 °C and 12 h of light/dark cycles on the Bloomington formulation of cornmeal agar medium.^[^ ^61^ ^]^

### Library Construction and Screening

To identity the potential protein(s) of *M. robertsii* that targets Spz, the fungal cDNA library was generated. The RNA samples were collected from the appressoria induced on the wings of black soldier fly (*Hermetia illucens*) for 24 h,^[^ ^15^ ^]^ and the hyphal body cells harvested from the hemolymph of silkworm larvae after injection with a spore suspension (20 µl each of 5 × 10^6^ conidia mL^−1^) for up to 60 h.^[^ ^47^ ^]^ An equal amount of each RNA sample was then pooled together for constructing a cDNA library in the pGADT7 vector, a service provided by the Shanghai Haike Biotechnology Company. For library screening, the *Spz* gene was cloned without its signal peptide (SP) region into the plasmid pGBKT7 and the obtained vector (200 ng) was used to transform the yeast strain Y2H Gold by following the standard protocols.^[^ ^27^ ^]^ The transformed yeast cells were spread on the SD‐Trp agar, and the positive clones were further transformed with the pGADT7 library DNA. Positive clones were obtained on the SD‐Trp‐Leu‐His‐Ala (TaKaRa) agar plus aureobasidin A (Yeasen) and/or X‐Gal (Yeasen).^[^ ^62^ ^]^ The plasmids were then extracted for sequencing and nine genes of *M. robertsii* were obtained (Table S1, Supporting Information).

### Gene Deletion and Overexpression in *M. Robertsii* and *B. Bassiana*

The verified Spz‐targeted genes *ETS1* and *ETS6* of *M. robertsii* were individually deleted by homologous recombination using the *A. tumefaciens*‐mediated transformation method.^[^ ^16^ ^]^ Briefly, the 5ʹ‐ and 3ʹ‐flanking regions (1–1.5 kb) of each gene were amplified by PCR using the genomic DNA as a template plus different primer pairs (Table S2, Supporting Information). The products were purified and subsequently inserted into the binary vector pDHt‐bar (conferring resistance to ammonium glufosinate)^[^ ^63^ ^]^ for *ETS1* and pDHt‐sur (conferring sulfonylurea resistance)^[^ ^25^ ^]^ for *ETS6*, respectively, to generate the knockout vectors pDHt‐bar‐ETS1 and pDHt‐sur‐ETS6. For further functional characterization of *ETS1*, the open reading frame of this gene was cloned into the vector pDHt‐Sur under the control of the *Tef*‐gene promoter of *M. robertsii* to generate the overexpression pSur‐ETS1 vector.^[^ ^47^ ^]^ The constructed vectors were used to individually transform the *M. robertsii* WT strain. The obtained mutants were verified by PCR using corresponding primers (Table S2, Supporting Information). The obtained Δ*ETS1* mutant was then used for transformation with the pDHt‐sur‐ETS6 plasmid to generate the double‐deletion mutant Δ*ETS1.6*. In addition, *BbETS1* was deleted in *B. bassiana* by following the same protocols. *ETS6* was also made under the control of the constitutive *GpdA* promoter to transform the WT strain of *B. bassiana*.^[^ ^64^ ^]^ At least three independent null mutants were selected during the deletion of each gene, and one stable strain was randomly selected for different experiments. The WT, single and double deletion mutant strains of *ETS1* and *ETS6* were first examined for appressorium induction on plastic hydrophobic surfaces and penetration assays using the cicada wings.^[^ ^15^ ^]^ Secretion features of the ETS1 and ETS6 SP regions were examined using a yeast‐secretion trap system as described.^[^ ^65^ ^]^

### Insect Bioassays

To assess the virulence of the WT and mutants of *M. robertsii*, it was conducted insect bioassays by topical infection or direct injection of the females or males of *D. melanogaster w^1118^* and *spz^rm7^* (two to three days post molting) as described before.^[^ ^27^ ^]^ The UAS‐Spz* and UAS‐*Tl^10B^* males were crossed with the virgins of Da‐Gal4 (*w*;*Da‐Gal4*;*Tub‐Gal80^ts^*), and F1 progeny females were also included in survival assays. Flies were immersed in each spore suspension containing 1 × 10^5^ conidia mL^−1^ in 0.05% Tween 20 for 30 s. For topical infections with *B. bassiana* strains, the spore suspensions were adjusted to 2 ×10^7^ conidia mL^−1^ in 0.05% Tween 20.^[^ ^66^ ^]^ The treated insects were kept at a high moisturizing condition (relative humidity > 95%) for 24 h and then maintained in growth chamber at 25 °C.^[^ ^27^ ^]^ For injection assays, female flies were individually injected with 20 nL of fungal spore suspension (5 × 10^6^ conidia mL^−1^), 50 nL of *Ecc15* (OD600 = 20) or *M. luteus* (OD600 = 20) cells suspended in sterile phosphate buffered saline (PBS, pH = 7.0) through thorax using a microinjector (Nanoject III, Drummond, Broomall).^[^ ^67^ ^]^ The insects treated with 0.05% Tween‐20 were used as mock controls. Insect mortality was recorded every 12 h.^[^ ^68^ ^]^ There were more than 70 flies used for each treatment, and the experiments were repeated at least twice. The difference in insect survival was determined by a log‐rank test using GraphPad Prism (ver. 10.1.1). The data shown were from the same batch of experiments.

### Yeast Two‐ and Three‐Hybrid Assays

To verify the potential interactions between Spz and other proteins, the bait vector pGBKT7‐Spz and the pGADT7 prey vector containing the individual prey genes without their SP coding sequences to co‐transform the yeast strain Y2H Gold by following the manufacturer's protocols was used (Takara Bio, 630 489). The plasmids pGADT7‐T and pGBKT7‐P53 were co‐transformed into yeast cells as a positive control, while co‐transforming pGADT7 and pGBKT7 into yeast cells was included as a negative control.^[^ ^16^ ^]^ Positive clones from the SD‐Leu‐Trp medium (TaKaRa) were transferred to the SD‐Leu‐Trp‐Ala‐His (TaKaRa) agar plus X‐gal to assess the positive (cell growth) or negative (no cell growth) interactions between bait and prey protein based on yeast cell survivals.^[^ ^62^ ^]^ To exclude the possibility of protein self‐activation, reciprocal Y2H analysis was performed by switching the bait and prey protein genes in the pGADT7 and pGBKT7 vectors, respectively. In addition, the *Spz* homologs of silkworm (BmSpz1, NP_001108066),^[^ ^33^ ^]^ mosquito *A. aegypti* (AaSpz1C, XP_021703650),^[^ ^34^ ^]^ and the spotted wing drosophila *D. suzukii* (DsSpz1, XP_036675611) were cloned from insect cDNA samples by excluding each SP region and used for interactive analysis with ETS1 and ETS6.

To determine whether ETS1 or ETS6 could block C106 dimerization and the interactions between SPE and Spz, and between C106 and Toll receptor, the Y3H analysis was performed by following the previous method.^[^ ^69^ ^]^ In brief, the *SPE*, *Spz/C106*, and alternative Toll ectodomains [Toll^28–397^ (Toll^EC^); Toll^28–668^ (Toll^5B^)]^[^ ^6^ ^]^ cDNAs were cloned into pGADT7, and their interactive partners were individually cloned into the multiple cloning site (MCS) I of pBridge vector (Clontech, TaKaRa). *ETS1* or *ETS6* was then cloned into the MCS II site of pBridge under the control of the *Met*‐repressible *pMET25* promoter. All plasmids were confirmed by PCR sequencing, and the pairwise pGADT7 and pBridge vectors were co‐transformed into the yeast AH109 strain. The transformed yeast cells were incubated on the SD‐Leu‐Trp‐Met medium (TaKaRa Bio) at 30 °C for 4 days. Individual colonies were then spotted onto the SD‐Trp‐Leu‐Met (to determine the correct transformation of vectors), SD‐Trp‐Leu‐His+Met plus 1 mM 3‐amino‐1,2,4‐triazole (3‐AT; to suppress the activation of effector genes), and SD‐Leu‐Trp‐His‐Met plus 1 mM 3‐AT (to activate effector genes) media (TaKaRa Bio), respectively. The inhibition of ETS1/ETS6 on fly immune protein interactions was determined by yeast survival assays and by using the substrate chlorophenol red‐β‐D‐galactoside (Sigma–Aldrich) to measure β‐galactosidase activity after induction on or in the SD‐Leu‐Trp‐His‐Met plus 1 mM 3‐AT medium based on protocols described in the yeast handbook PT3024‐1 (Clontech).

### Verification of Protein Interactions

To further verify the interactions between proteins, the heterologous expressions of GST, GST‐ETS1, and GST‐ETS6; maltose‐binding protein (MBP) and its tagged MBP‐Spz, and C106‐His (6×) was performed in the *E. coli* BL21(DE3) cells by incubation at 18 °C, and induction using 0.2 mM isopropyl ß‐D‐1‐thiogalactopyranoside for 12 h. After incubation, bacterial cells were harvested by centrifugation and re‐suspended in buffer (50 mM Tris‐HCl, pH 8.0; 150 mM NaCl; 1 mM PMSF) for cell disruption using the OS Shot Cell Disrupter (Constant Systems, UK). The mutagenized ETS6 derivatives mentioned above were also expressed for IP analysis with C106. Protein purifications and pull‐down analysis were conducted as we described before.^[^ ^27^ ^]^ In brief, the GST‐ and His‐tagged proteins were purified by affinity chromatography using Glutathione Sepharose (TransGen Biotech) and Ni‐NAT Superflow Agarose columns (SMART Life Sciences), respectively. The MBP‐Spz was purified using the amylose resin columns (Sangon Biotech). The HRP (horseradish peroxidase)‐conjugated goat anti‐mouse/rabbit IgG secondary antibodies (Epizyme) were used, and membranes were treated with the Omni‐ECL (Epizyme) substrate solution before photographing using a Chemiluminescent Imaging System (Tanon). Protein samples were also collected from *w^1118^* flies after topically infected with the WT *M. robertsii* for 60 h, and used for Co‐IP analysis using the purified GST‐ETS1/ETS6 proteins, respectively. The input and output samples were blotted with the anti‐GST and anti‐C106 antibodies, respectively.

### Proteolytic Degradation Assays of Spz and C106 by ETS1

As a confirmed target of *Drosophila* Spz/C106, it was curious to know whether ETS1 could degrade this antifungal Toll ligand. Considering the MP activity requires the metal ion cofactor,^[^ ^70^ ^]^ the proteolytic reaction system (50 µL) including ETS1 (at a final concentration of 1 µg µL^−1^), C106 (2 µg µL^−1^) and 1 mM of ZnCl_2_, CoCl_2_, CaCl_2_, CoCl_2_/CaCl_2_, and ZnCl_2_/CaCl_2_ in 50 mM Tris‐HCl buffer (pH = 8) was prepared. C106 with only cations and the system without any metal ion were included as controls. The reactions were kept at 37 °C for 5 h before examined with the analysis of sodium dodecyl‐sulfate polyacrylamide gel electrophoresis (SDS‐PAGE). After confirming that CoCl_2_ and CaCl_2_ were the most optimal cofactors, the degradation of Spz (2 µg µL^−1^) and C106 (2 µg µL^−1^) by ETS1 (2 µg µL^−1^) was then examined over a time course of 0–4 h. In addition, DsSpz1‐C104, AaSpz1C‐C97, and BmSpz1‐107 were expressed, purified, and used for ETS1 degradation assays. The BSA protein (2 µg µL^−1^) was also included as a substrate control of ETS1 (2 µg µL^−1^). After reaction for different durations, samples were added with the SDS‐PAGE loading buffer, boiled and subjected to protein gel profiling. Individual proteins were added to the reaction buffer simultaneously and incubated until the reaction was complete before being loaded as controls. The experiments were repeated twice. Protein gels were stained using a Coomassie Brilliant Blue R‐250 (Sigma–Aldrich) dye, and the banding intensity of the target proteins was quantified using ImageJ software (ver. 1.53).^[^ ^71^ ^]^

### Protein Structure Prediction and Mutagenesis of ETS6

Based on the known structures of the *D. melanogaster* and *A. aegypti* Toll receptor ligands,^[^ ^6^, ^34^ ^]^ Spz homologs reported in different invertebrates were selected for structure predictions using AlphalFold.^[^ ^72^ ^]^ The mature C‐terminal amino acids were used as reported or deduced from different animals, including the spotted wing drosophila *D. suzukii* (DsSpz1, XP_036675611), silkworm *B. mori* (BmSpz1, NP_001108066),^[^ ^33^ ^]^ mosquito *A. aegypti* (AaSpz1C, XP_021703650),^[^ ^34^ ^]^ red flour beetle *Tribolium castaneum* (TcSpz1, XP_008201187),^[^ ^73^ ^]^ pea aphid *Acyrthosiphon pisum* (ApSpz1, NP_001153590),^[^ ^74^ ^]^ deer tick *Ixodes scapularis* (IsSpz1, EEC16960),^[^ ^74^ ^]^ and water flea *Daphnia pulex* (DpSpz1, EFX85931).^[^ ^75^ ^]^ Pairwise structure alignments were performed by reference to the *Drosophila* C106‐dimer structure to estimate the TM scores.^[^ ^38^ ^]^

The ETS6‐C106 complex structure was predicted using AlphaFold multimer (Ver. 2.3.2) following instructions.^[^ ^76^ ^]^ The predicted structure of ETS6 (AlphalFold ID: AF‐A0A0A1V283‐F1) and X‐ray structure of C106 dimer (PDB: 3E07) were downloaded from Uniprot, and protein structure models were generated using PyMol (Ver. 2.5.5, Schrödinger, LLC). The Predicted Aligned Error (PAE) of the structure complex was processed using the PAE Viewer.^[^ ^77^ ^]^ To verify the function of the three‐block structure of ETS6, truncated *ETS6* derivatives were generated by PCR or fusion PCR using its cDNA as a template and different primers (Table S2, Supporting Information), including the generations of ETS6.B1, ETS6.B2, ETS6.B3, ETS6.B1.2, ETS6.B2.3, and ETS6.B1.3. Different fragments were used for Y2H, Y3H, protein expressions, and co‐IP analyses.

### Phylogenetic Analysis

The homologous protein sequences of ETS1 (MrM28‐1) and ETS6 were retrieved from different ascomycete fungi, and aligned with CLUSTAL X.^[^ ^78^ ^]^ Phylogenetic relationships of these proteins were generated with the program MEGA11.^[^ ^79^ ^]^ The neighbor‐joining trees were referred using the Jone‐Taylor‐Thornton model with the partial deletion of gap/missing data and 500 bootstrap replicates.

### Generation of the ETS1 and ETS6 Transgenic Drosophila

The cDNA sequences of *ETS1* and *ETS6* were cloned into the plasmid pUAST‐attB, and the vectors were used to inject the fly embryos at the National Drosophila Resource Center of China (http://ndrcc.sibcb.ac.cn/ndrcc/). The isogenic male flies were crossed with the virgins of the Da‐Gal4 (*w*; *Da‐Gal4*;*Tub‐Gal80^ts^*) flies. The F1 progeny female or male flies were maintained at 29 °C for three days before use for experiments together with the Da>*mCherry* flies as a control.

### Gene and Protein Expression Analysis

To investigate the pattern of the putative target gene expressions in *M. robertsii*, different samples were collected for RNA extractions. The samples included the mycelia collected from the three‐day‐old SDB cultures, conidial spores harvested from the 2‐week‐old PDA plates, appressoria induced on cicada wings for 24 h, and hyphal body cells harvested from the hemolymph of the wax moth larvae after injection through the second proleg with the WT spores of *M. robertsii* (10 µL each of 1 × 10^6^ conidia mL^−1^) for three days.^[^ ^80^ ^]^ To determine the expression of AMP genes in *Drosophila* after fungal infections, flies were immersed for 30 sec in a conidial suspension (5 × 10^5^ conidia mL^−1^) of *M. robertsii*, and collected at different time points for RNA extraction. Da>*ETS1*, Da>*ETS6*, and Da>*mCherry* flies injected with *Ecc15* or *M. luteus* cells (50 nL each of OD600 = 20) were also used for RNA extraction. Total RNA was extracted using the TransZol‐Up plus RNA Kit (Transgen Biotech), and cDNA was synthesized using the HiScript III RT Reagent Kit (Vazyme). The qRT‐PCR analysis was performed using an SYBR mix (Yeasen) and a PikoReal Real‐Time PCR System (Thermo, N11471). Target gene expression ratio was normalized to either the fungal (*Metarhizium*) or *Drosophila* reference gene. The primers used for the target genes and the reference β‐tubulin gene *MrTub* of *M. robertsii* and *Rp49* of *D. melanogaster* were listed in Table S2 (Supporting Information).

After the topical infection of Da>*ETS1*, Da>*ETS6*, and Da>*mCherry* flies with the WT spores of *M. robertsii* for different durations, insects (100 each: 50 females and 50 males) were used for hemolymph collection using Zymo‐Spin IC Columns (Zymo Research) by centrifugation.^[^ ^81^ ^]^ Each sample was diluted two times with the phosphate saline solution before being loaded for protein gel analysis and Western blotting using the anti‐C106 antibody generated before.^[^ ^24^ ^]^ All samples were also subjected to parallel SDS‐PAGE analysis and Coomassie brilliant blue staining, which served as loading controls.

### Statistical Analysis

Statistical analyses were performed using data from a minimum of three independent experiments. Values are presented as the mean ± standard error of the mean (SEM) for survival data or the mean ± standard deviation (SD) for bar graphs. Statistical comparisons between groups were conducted using two tailed Student's *t‐*test (for normally distributed parametric data) or one‐way analysis of variance (ANOVA, for multiple distributed parametric data). The Kaplan‐Meier analyses followed by log‐rank tests were conducted to compare the insect survival kinetics between different challenges. Data analysis utilized the software GraphPad Prism (ver. 10.2, GraphPad Software Inc., San Diego, CA). Statistical significance was set at *p <* 0.05.

## Conflict of Interest

The authors declare no conflict of interest.

## Author Contributions

C.W. conceived and designed the project, analyzed the data and wrote the manuscript. S.S. performed experiments and analyzed the data. S.L., Y.L., J. S., and H.W. performed yeast library screening and insect bioassays. G.‐Q.F. performed protein structure prediction.

## Supporting information



Supporting Information

## Data Availability

The data that support the findings of this study are available in the supplementary material of this article.
