# Supplementary material for: Dual Disruption of the Immune Cytokine Spätzle Facilitates Fungal Infection of Diverse Insect Hosts
Source: Adv Sci (Weinh). 2025 Oct 20;13(1):e13075. doi: 10.1002/advs.202513075 (PMC12767022; doi:10.1002/advs.202513075)
Supplement: Supplementary file 1 — Supporting Information [file ADVS-13-e13075-s001.docx]

**Supporting information**

**Dual Disruption of the Immune Cytokine Spätzle Facilitates Fungal Infection of Diverse Insect Hosts**

*Shuangxiu Song^1^, Shiqin Li^1,3^, Yujuan Luo^1,3^, Dongxiang Wei^1,2^, Junmei Shang^1^, Hongyun Wu^1,3^, Gangqi Fang^1^, and Chengshu Wang^1,2,3^**

^1^Key Laboratory of Insect Developmental and Evolutionary Biology, CAS Center for Excellence in Molecular Plant Sciences, Shanghai Institute of Plant Physiology and Ecology, Chinese Academy of Sciences, Shanghai 200032, China.

^2^CAS Center for Excellence in Biotic Interactions, University of Chinese Academy of Sciences, Beijing 100049, China.

^3^School of Life Science and Technology, ShanghaiTech University, Shanghai 201210, China.

*To whom correspondence may be addressed: [wangcs@sippe.ac.cn](mailto:wangcs@sippe.ac.cn)


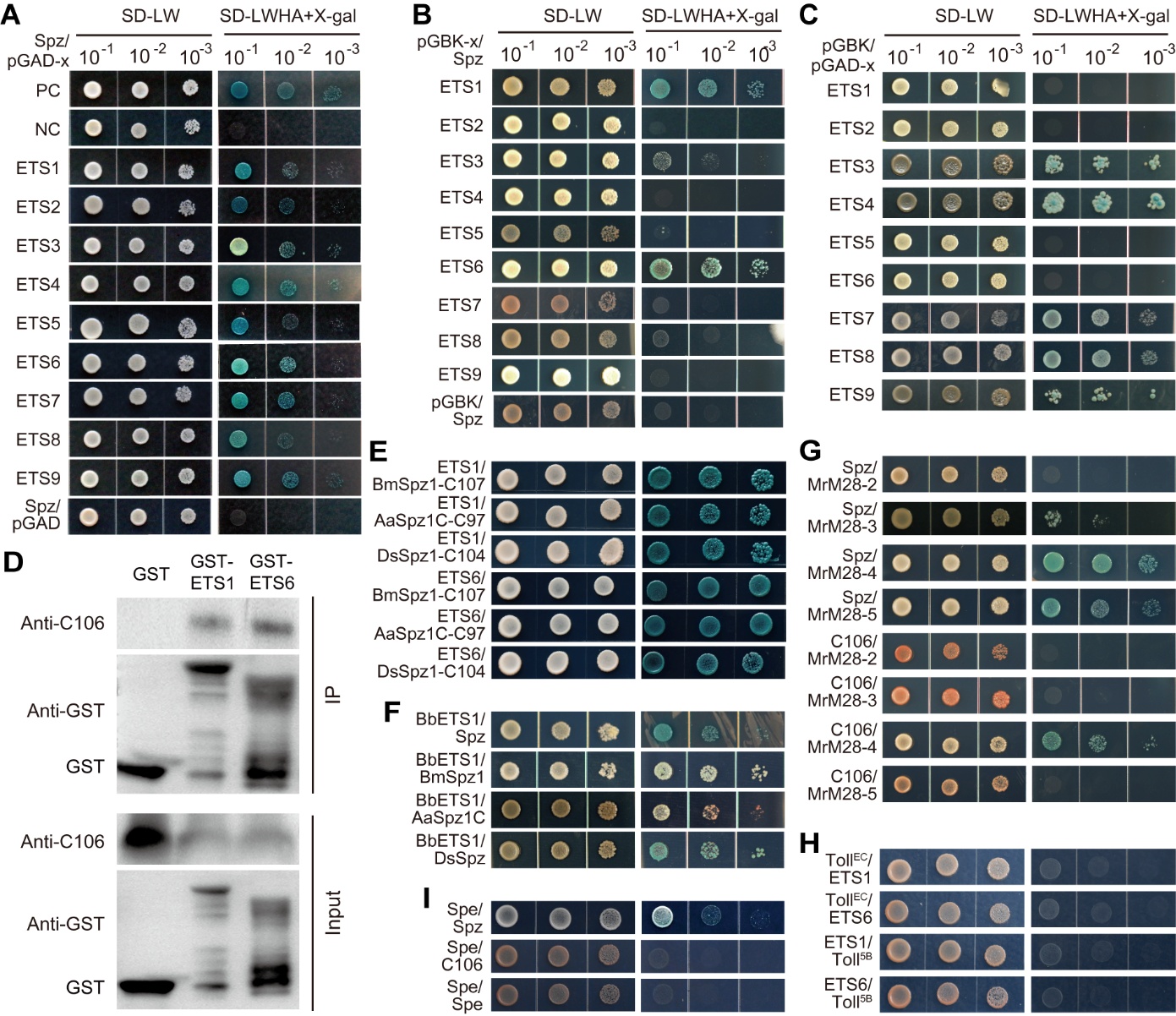


**Figure S1.** Verification of protein interactions. A,B) Verification of protein interactions by using the *Metarhizium* proteins as either prey (A) or bait (B) in Y2H analysis. PC, positive control; NC, negative control. The synthetic dropout (SD) agar lacking Leu and Trp (LW) was used to show the yeast cells being successfully transformed with the bait/prey plasmids while the inoculations on the SD medium lacking Leu, Trp, His and Ala (LWHA) plus X-gal were for verification of the positive (cell growth) or negative (no cell growth) interaction between bait and prey proteins. C) Verification of the self-activation of fungal proteins in Y2H analysis. D) Co-IP analysis confirms the successful pulling-down of C106 by GST-ETS1/ETS6 from the *Metarhizium*-challenged *Drosophila* protein samples. GST was used as a control that could not bind C106. IP, immune-precipitation. E) Both ETS1 and ETS6 individually target the deduced C-termini of BmSpz1 (*B. mori*), AaSpz1C (*A. aegypti*), and DsSpz (*D. suzukii*). F) Verification of the *B. bassiana* ETS1 in interacting with the Spz homologs of *D. melanogaster* (Spz), *B. mori* (BmSpz1), *A. aegypti* (AaSpz1C) and *D. suzukii* (DsSpz). G) Verification of the paralogous ETS1 proteins in interacting or not with the *Drosophila* Spz and C106. H) Both ETS1 and ETS6 cannot interact with the ectodomains of the *Drosophila* Toll receptor. Toll^EC^, the ectodomain region of Toll (28-397 aa); Toll^5B^, the extracellular region 28-668 aa of Toll. I) Spe targets Spz but not C106, and there is no Spe self-interaction.


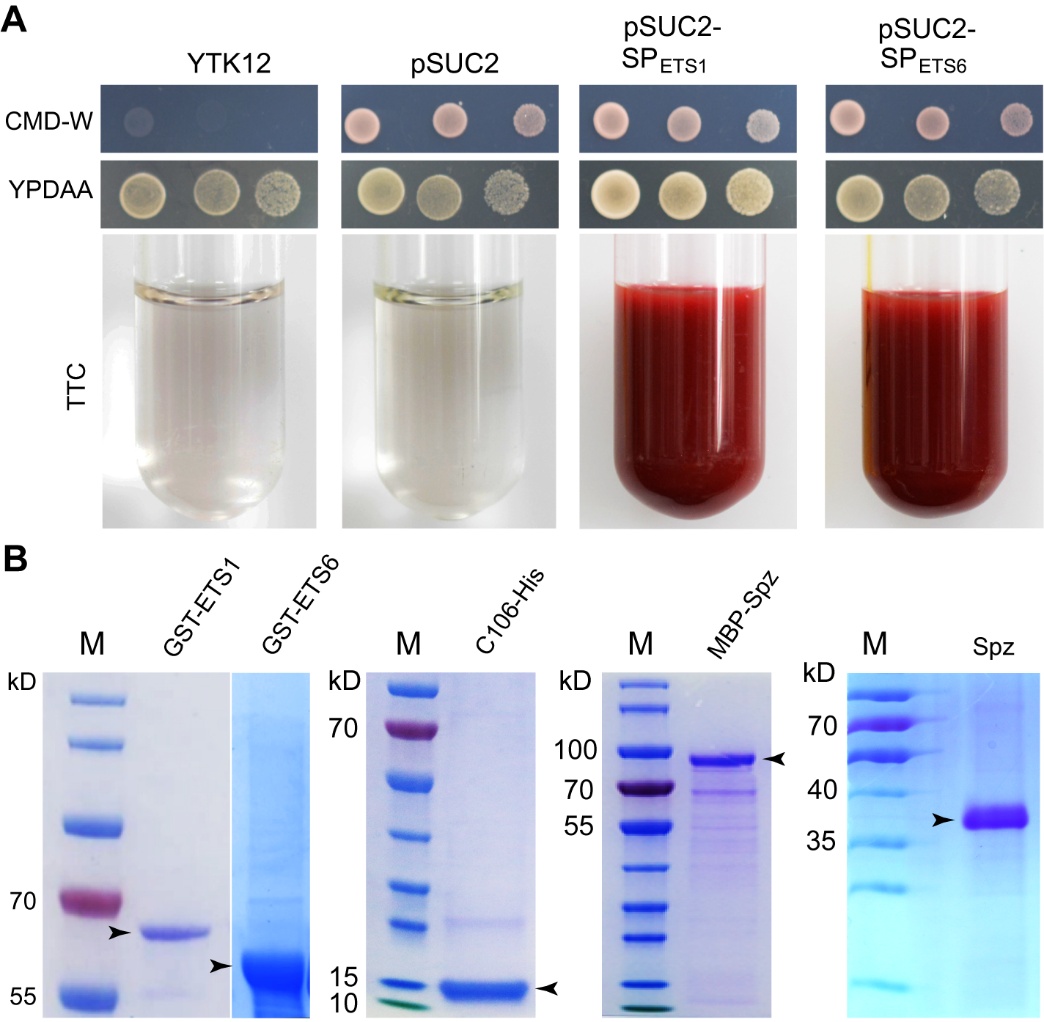


**Figure S2.** Protein secretion and expression analysis. A) Verification of the secretion nature of ETS1 and ETS6 using a yeast secretion-trap system. The complete dropout medium without Trp (CMD-W) was used to select the yeast strain YTK12 carrying the pSUC2 plasmid, and the yeast extract, peptone and raffinose and antimycin A (YPRAA) medium was used to indicate the reduction of tetrazolium chloride (TTC) to red formazan by the secreted invertase mediated by the signal peptides of ETS1 (SP_ETS1_) and ETS6 (SP_ETS6_). B) SDS-PAGE analysis of the heterogeneously expressed and purified proteins. Target proteins are arrowed. GST, glutathione S-transferase; MBP, maltose-binding-protein.


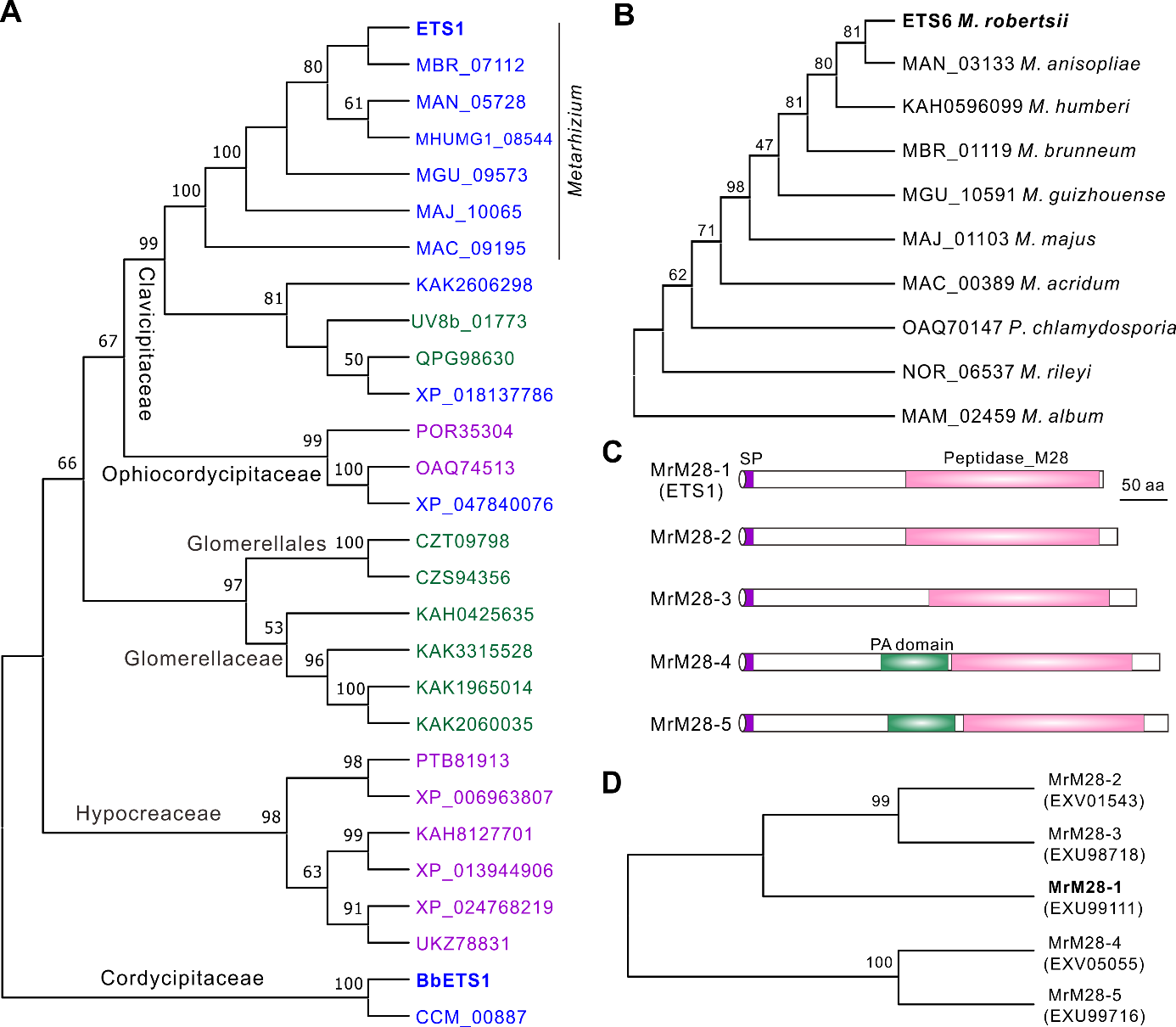


**Figure S3.** Protein evolution analyses. A) The consensus evolutionary relationship of the ETS1-like metalloproteases from different fungi. The neighbor-joining (NJ) tree was generated using a Jones-Taylor-Thornton (JTT) model and 500 bootstrap replicates. Sequences labeled in blue are from entomopathogenic fungi; those in green are from plant pathogenic fungi or endophytes; and those in purple are from mycoparasitic fungi. B) Evolutionary analysis of the ETS6-like proteins being present in different *Metarhizium* species. The nematophagous fungus *P. chlamydosporia* also contains an ETS6 homolog. The consensus NJ tree was generated using a JTT model and 500 bootstrap replicates. C) Schematic structures of five secreted M28 domain-containing proteins encoded by *M. robertsii*. SP, signal peptide; PA, protease associated. D) The consensus neighbor-joining phylogenetic relationships of five M28 metalloproteases encoded by *M. robertsii*.


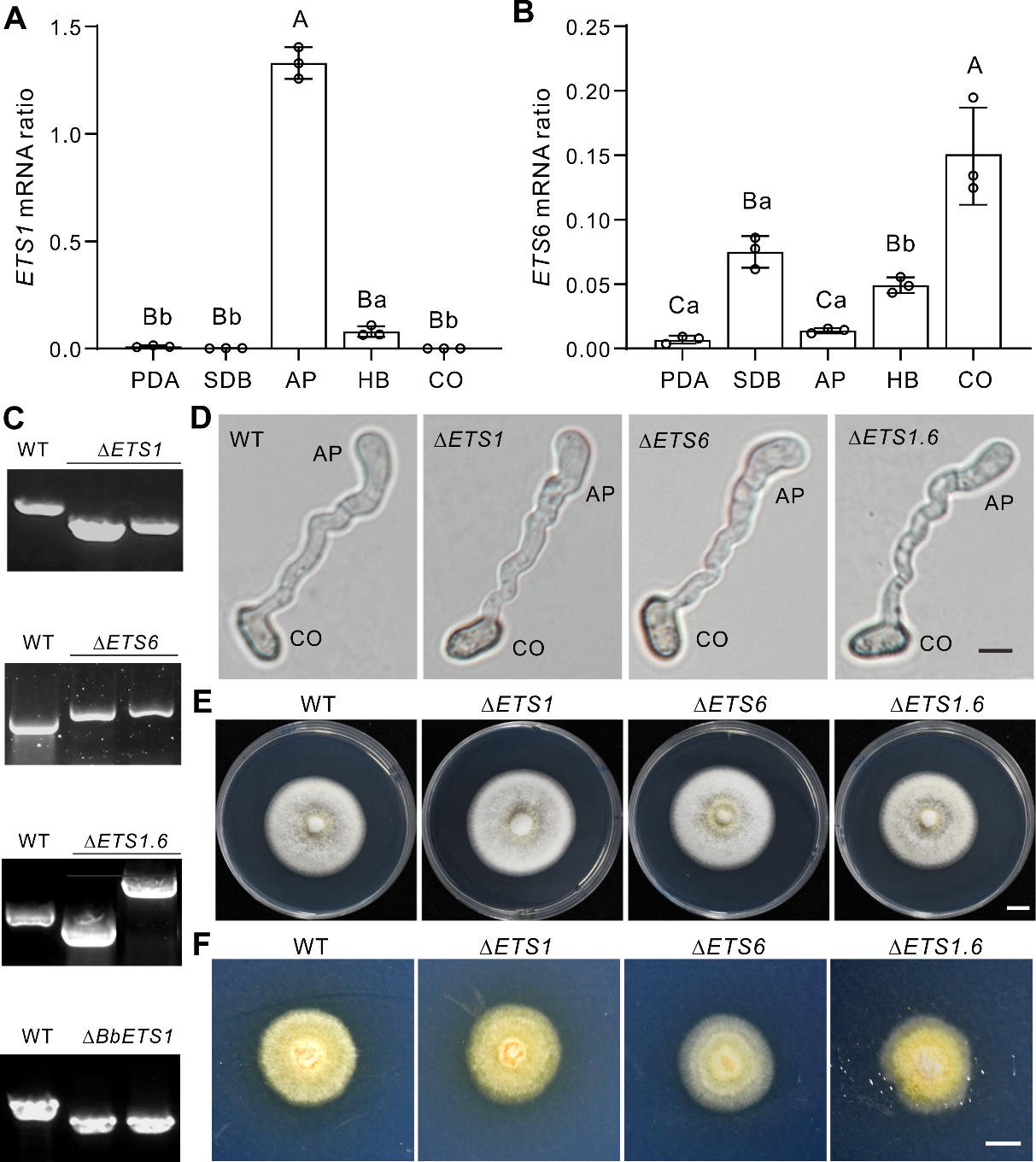


**Figure S4.** Phenotyping of gene expression and fungal mutants, fly survival and AMP gene expression analysis. A,B) qPCR analysis of *ETS1* (A) and *ETS6* (B) gene expressions in *M. robertsii* growing at different conditions or stages. PDA, conidia harvested from the 14 d old PDA plates; SDB, mycelia harvested from the 3 d old SDB broth; AP, appressoria induced on the cicada wings for 24 h; HB, hyphal body cells harvested from the body cavity of the wax moth larvae 3 d post injection with the *M. robertsii* spores; CO, conidia harvested from the mycosed wax moth larvae. There were three independent repeats for each sample. Data are the mean ± SD. One-way ANOVA followed by Tukey’s test: different capital letters, *p <*0.01; different lower letters, *p <*0.05. C) PCR verification of the individual and double deletion of *ETS1*/*ETS6* genes in *M. robertsii* and *BbETS1* in *B. bassiana*. The primers flanking the drug-resistance genes were used for PCR amplifications. D) Deletion of *ETS1* and *ETS6* in *M. robertsii* had no obvious negative effect on appressorium (AP) formation. The conidial spores were inoculated on a hydrophobic surface for 18 h. CO, conidium. Bar, 5 μm. E,F) Individual or double deletion of *ETS1*/*ETS6* genes did not affect fungal growth on PDA (E) and penetration of cicada wings (F). Bar, 1 cm. Spore suspension (3 μl) was inoculated on PDA for 10 days or the middle of cicada wings for four days. The wings were carefully removed and the plates were kept for incubation for five additional days before imaging.


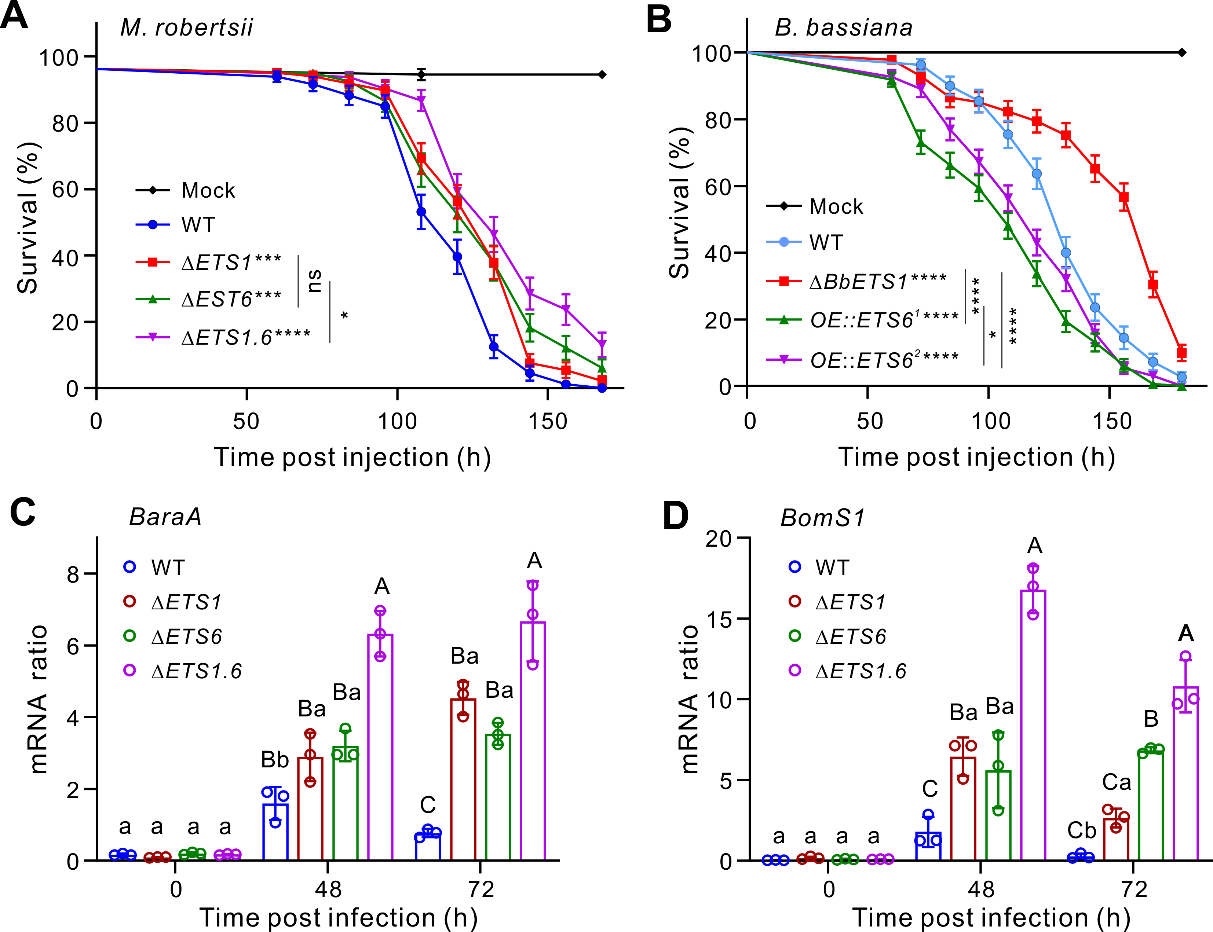


**Figure S5.** Fly survival and AMP gene expression analysis. A) Survival of *D. melanogaster* females injected with the spores of the WT and mutant strains of *M. robertsii*. B) Survival of *D. melanogaster* females after topical infections with the WT and mutant strains of *B. bassiana*. C,D) RT-qPCR analysis showing the differential expression of *BaraA* (C) and *BomS1* (D) genes in the female flies after topical infections with WT and mutant strains of *M. robertsii* for different times. Panels A and B: There are more than 70 flies used for each treatment. Plotted values are the mean ± SEM, obtained by Kaplan-Meier analysis. Log-rank: **p <*0.05; ****p <*0.001; *****p <*0.0001. ns, not significant. Panels C and D: There were three independent repeats for each sample. Data are the mean ± SD. One-way ANOVA followed by Tukey’s test was conducted between samples examined at the same time: different capital letters, *p <*0.01; different lower letters, *p <*0.05.


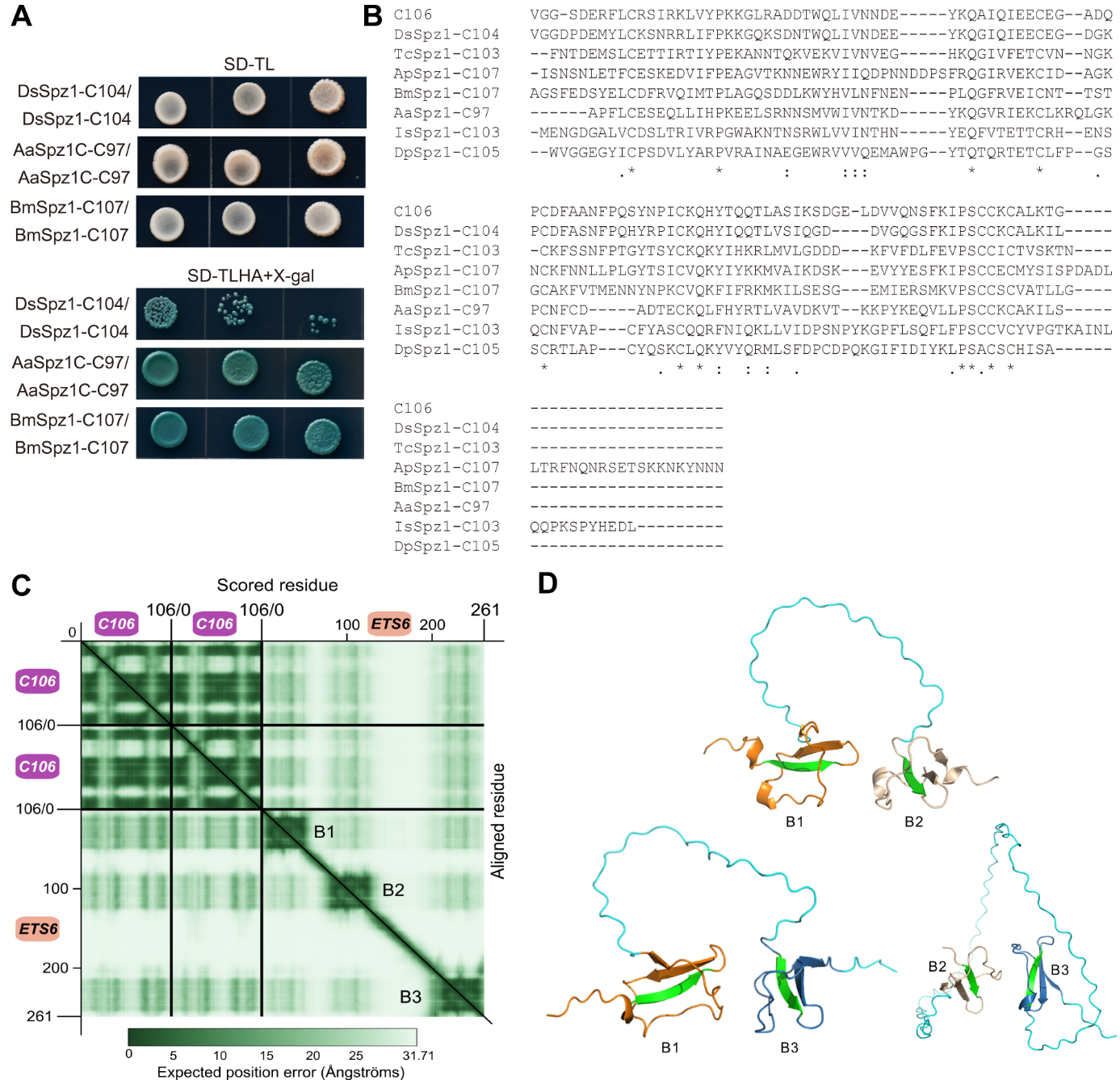


**Figure S6.** Protein interaction, sequence alignment and structuring analyses. A) Y2H analysis shows the self-interaction of DsSpz1-C104, AaSpz1C-C97, and BmSpz1-C107. B) Sequence alignment shows sequence divergence among different Toll receptor ligands. Alignment was performed using Clustal X. Different ligands are DsSpz1-C104, spotted-wing drosophila *D. suzukii*; BmSpz1-C107, silkworm *B. mori*; TcSpz1-C103, red flour beetle *Tribolium castaneum*; ApSpz1-C107, pea aphid *Acyrthosiphon pisum*; IsSpz1-C103, deer tick *Ixodes scapularis* and DpSpz1-C105, water flea *Daphnia pulex*. C) PAE (Predicted Aligned Error) viewing of the ETS6-C106 complex structure. D) Structure prediction of the truncated ETS6 isoforms B1.2, B1.3, and B2.3 using AlphaFold3. The β-sheets in green in each block correspond to the CCCCD/N motifs.


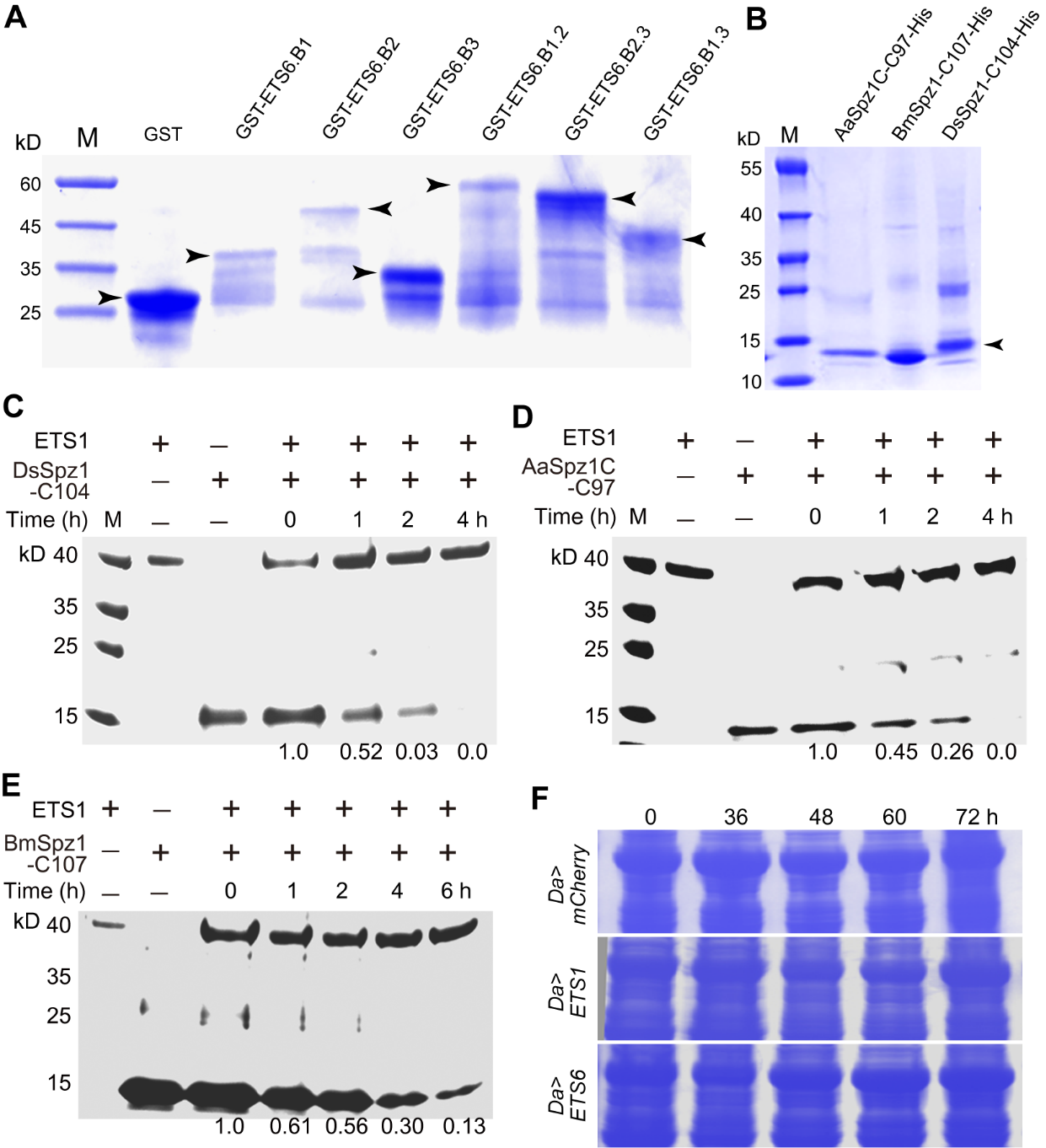


**Figure S7.** Protein expression and degradation analysis. A,B) Expression and purification of the truncated ETS6 fragments (A, arrowed) and C-terminal ligands of different insects (B, arrowed). C-E) Proteolytic degradation of DsSpz1-C104 (C), AaSpz1C-97 (D), and BmSpz1-C107 (E) by ETS1 for different times. Each reaction system (50 μl) contained the final concentration of 2 μg μL^−1^ ETS1, and 2 μg μL^−1^ each ligand. Band intensities were quantified using ImageJ, and the values labeled below each band were calculated by reference to the band value labeled as 1.0 in each panel. F) Gel analysis of hemolymph proteins isolated from different female flies after topical challenge with the WT strain of *M. robertsii* for different times.


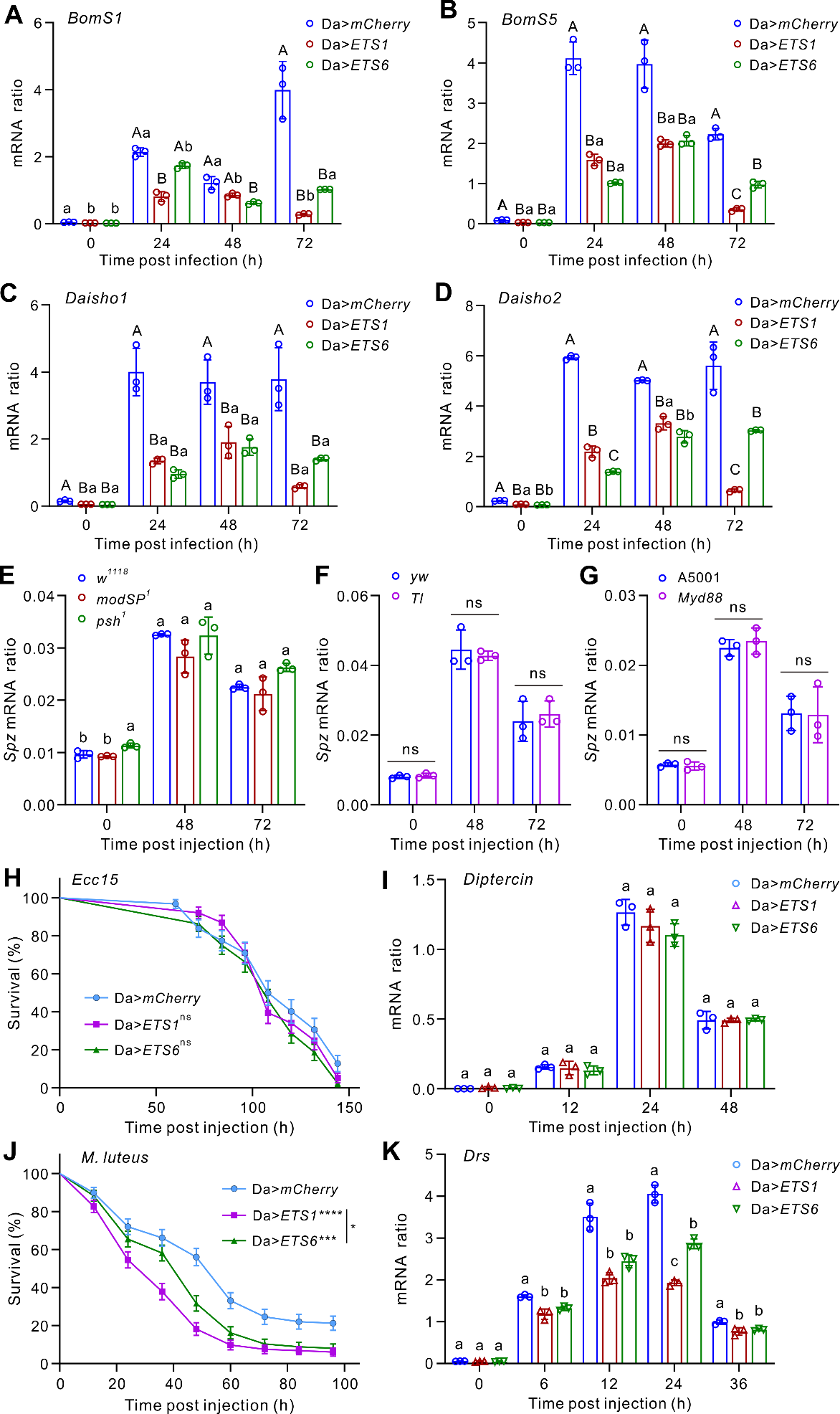


**Figure S8.** Gene expressions and fly survival assays against bacteria. A-D) RT-qPCR analysis showing the reduced expression of the antifungal genes *BomS1* (A), *BomS5* (B), *Daisho1* (C), and *Daisho2* (D) in the *ETS1-* and *ETS6-*transgenic flies when compared with the mock control after topical infection with the WT strain of *M. robertsii* for different times. E-G) RT-qPCR analysis of *Spz* expression in the WT and mutant flies of *ModSp^1^* and *Psh^1^* (E), *Tl* (F) and *MyD88* (G) after topical infection with the WT strain of *M. robertsii* for different times. H) Fly survivals after injection with *Erwinia carotovora carotovora* 15 (*Ecc15*). I) No difference in *Diptericin* expression in the *ETS1-* and *ETS6-*transgenic flies when compared with the mock control after injection with *Ecc15* cells for different times. J) Fly survivals after injection with *M. luteus*. I) Differential *Drs* expression in the *ETS1-* and *ETS6-*transgenic flies after injection with *M. luteus* cells for different times. Panels A-G, I and K: There were three independent repeats for each sample. Data are the mean ± SD. Panels A-E, I and K: One-way ANOVA followed by Tukey’s test was conducted between samples examined at the same time: different capital letters, *p <*0.01; different lower letters, *p <*0.05. Panels F and G: two tailed Student’s *t*-test: ns, not significant. Panels H and J: There are more than 70 flies used for each treatment. Plotted vales are the mean ± SEM, obtained by Kaplan-Meier analysis. Log-rank test between transgenic and control flies: ****p* < 0.001; *****p* < 0.0001; ns, not significant.

**Table S1.** Putative target genes of *M. robertsii* identified by screening with the *Drosophila* Spz as a bait protein.

| **Gene** | **Accession** | **Length (aa)** | **SP region (aa)** | **Annotation** |
| --- | --- | --- | --- | --- |
| *ETS1* | EXU99111 | 372 | 1 to 20 | Peptidase family M28 protein |
| *ETS2* | EXV00235 | 125 | 1 to 28 | Hypothetical protein |
| *ETS3* | EXU94976 | 256 | 1 to 21 | Peptidase S1 domain protein |
| *ETS4* | EXV03291 | 299 | 1 to 18 | Peptidase M43 family protein |
| *ETS5* | EXV04324 | 388 | 1 to 15 | Peptidase S8 family protein Pr1A |
| *ETS6* | EXV03935 | 279 | 1 to 19 | Hypothetical protein |
| *ETS7* | EXU99948 | 205 | 1 to 24 | Hypothetical protein |
| *ETS8* | EXU97964 | 145 | 1 to 17 | Hypothetical protein |
| *ETS9* | EXV01861 | 255 | 1 to 21 | Peptidase S1 domain protein |

**Table S2.** Primers used in this study.

| **Primers** | **5'-3' Primer sequences** |
| --- | --- |
| **Gene deletion and overexpression** | |
| ETS1-DF | CACCGAGATCTGATGATGACGGCCCTGATAGGGGAC |
| ETS1-DR | CCGCTCTAGAACTAGTTTGGCGAGTTTAGCATTTATAGCGTT |
| ETS1-UF | CTGCAGCCCGGGGGATCCGCCCCATCGCGCATAGTC |
| ETS1-UR | CAATATCATCTTCTGTCGACTGAGCAAGTTCTCTTCTCCACACCAT |
| ETS1-VF | ATCTATGTGTGATGAGGGGGCG |
| ETS1-VR | AACGCTTCCTTTTTCACACCACA |
| OE-ETS1-F | ACACAACCGTCAACTCTAGAATGCCTTCCATGTCCAAGCTC |
| OE-ETS1-R | GCGGTGGCGGCCGCTCTAGATTAGATGTACGACGCTTCCAGGAGG |
| OE-ETS1-VF | ACACAACCGTCAACTCTAGA |
| OE-ETS1-VR | GCGGTGGCGGCCGCTCTAGA |
| ETS6-UF | CTGCAGCCCGGGGGATCCGAGTACTGCACACACGTACATGTG |
| ETS6-UR | CAATATCATCTTCTGTCGACCCACACACCACCATCCTCACACA |
| ETS6-DF | CACCGAGATCTGATGAGTCCGGTGCTGAATATGACTTGTTG |
| ETS6-DR | CCGCTCTAGAACTAGTCACCAGACATCACATTCACGACC |
| ETS6-VF | CTTGTACGCCGTGTGTGAGGATG |
| ETS6-VR | GACCAACAAGTCATATTCAGCACCG |
| OE-ETS6-F | ACACAACCGTCAACTCTAGA ATGCGCTTTACTTGGATTGCTACC |
| OE-ETS6-R | GCGGTGGCGGCCGCTCTAGATTAGATGTTGGTGCACACAGGAGG |
| OE-ETS6-VF | CGTATCTGGTAAGCTTTGACCTCC |
| OE-ETS6-VR | TTAGATGTTGGTGCACACAGG |
| surETS6-DF | CTCTCACGTCGACTAGTTCTAGACAAATCGAGGCATAACGCGTC |
| surETS6-DR | CCGCGGTGGCGGCCGCTCTAGACTCACGATGGCGTCGTACATG |
| surETS6-UF | ATTCCTGCAGCCCGGGGGATCCCATGAAGGCCAATATGTTGATGTC |
| surETS6-UR | GGCGTTGGCACGTCGACGGATCCGCCATTCACCATCGTACCGTCAC |
| surETS6-VF | CTACTACCTTACCCATGACAC |
| surETS6-VR | CATCTCTGCAAGTCGTTGGAAC |
| BbETS1DF | CACCGAGATCTGATGACTACATCCAGGGCACTCTCGA |
| BbETS1DR | CCGCTCTAGAACTAGTCGTTCGGGGTGCAATTCCC |
| BbETS1UF | CTGCAGCCCGGGGGATCCGTGGTATCCGAAACTAGCTAGGG |
| BbETS1UR | CAATATCATCTTCTGTCGACCACTGCACCTACTATAAATGGCATG |
| BbETS1VF | GTGCGACGTTGTACCCAACT |
| BbETS1VR | GTCAGGCCAGGATCGACAAAG |
| BbOE-ETS6-F | TCAATAACAACTAGTTCTAGAATGCGCTTTACTTGGATTGCTAC |
| BbOE-ETS6-R | CGCGGTGGCGGCCGCTCTAGATTAGATGTTGGTGCACACAGGA |
| **Yeast two-hybrid analysis** | |
| BK-Spz-F | GAGGAGGACCTGCATATGATGAAGGAGTATGAACGTATCATCAAAG |
| BK-Spz-C106F | GAGGAGGACCTGCATATGATGGTTGGTGGCTCAGACGAGCG |
| BK-Spz-R | CGCTGCAGGTCGACGGATCCTCACCCAGTCTTCAACGCGCAC |
| BK-SPE-F | GAGGAGGACCTGCATATGATTTTTGGTGGTACAAATACCACCC |
| BK-SPE-R | CGCTGCAGGTCGACGGATCCTCACGGCTCCAGCTTCTGTTTAATCC |
| BK-BbETS1-F | GAGGAGGACCTGCATATGCGCTTTATCGAAAAAGGCACCGCCA |
| BK-BbETS1-R | CGCTGCAGGTCGACGGATCCTTACAGCTCGAGATTGTCAAGGC |
| BK-ETS1-F | GAGGAGGACCTGCATATGCCTGCTGGTCTGACCCGTC |
| BK-ETS1-R | CGCTGCAGGTCGACGGATCCTCAAATATAGCTGGCTTCCAGCAGATAAC |
| BK-ETS6-F | GAGGAGGACCTGCATATGGCCCAGCCTCCCAGAGACTCG |
| BK-ETS6-R | CGCTGCAGGTCGACGGATCCTTAGATGTTGGTGCACACAGGA |
| AD-BmSpz1-F | CTCGAGCTCGATGGATCCTCATTAACCGAGTAGCGTGGCAACAC |
| AD-BmSpz1-R | CCAGATTACGCTCATATGTCAATATCGGCCTACAA |
| AD-AaSpz1-F | CCAGATTACGCTCATATGTCATCGGCCATCCCGCTGATAC |
| AD-AaSpz1-R | CTCGAGCTCGATGGATCCTTAGAATCCCTTGAATCGGACCACC |
| AD-DsSpz1F | CCAGATTACGCTCATATGAAGGAATATGATGGTATCATCAAAGAG |
| AD-DsSpz1R | CTCGAGCTCGATGGATCCTCACCCGTCTTCAACGCG |
| AD-ETS1-2F | CCAGATTACGCTCATATGCGCTTCGTTGAGCTCATGGAG |
| AD-ETS1-2R | CTCGAGCTCGATGGATCCCTATAGTTCGCCGTCACCCTCGAC |
| AD-ETS1-4F | CCAGATTACGCTCATATGACCGAGCTTCTCACCCCGGACAA |
| AD-ETS1-4R | CTCGAGCTCGATGGATCCCTAATACAGACTATCGCCCGAGCCG |
| AD-ETS1-5F | CCAGATTACGCTCATATGCTGCAGCTCCCTCTGCAGCG |
| AD-ETS1-5R | CTCGAGCTCGATGGATCCCTACGAACGACAAAACTGCGGCG |
| AD-ETS1-3F | CCAGATTACGCTCATATGTCTCGAGACGCATCCCGAGAC |
| AD-ETS1-3R | CTCGAGCTCGATGGATCCTCACCTCCATTTGTTGTCCCAGC |
| AD-ETS9-SP-F | CCAGATTACGCTCATATGGCGCCGGCCAAGAATGGCAAGT |
| AD-ETS9F | CCAGATTACGCTCATATGATGGCCTCCAAGGCAGCTCTC |
| AD-ETS9R | CTCGAGCTCGATGGATCCCTACAGGTACTGCTCGATAAAGCTG |
| AD-ETS8-SP-F | CCAGATTACGCTCATATGATTGAGCCCCGGGAGGGTCA |
| AD-ETS8R | CTCGAGCTCGATGGATCCTTACAAGTCCTCGCAGGAGTC |
| AD-ETS4-SP-F | CCAGATTACGCTCATATGGTCGACAAACCCCTTGACGTG |
| AD-ETS4F | CCAGATTACGCTCATATGATGCTTCACAGTCTTGCAATCTCAAG |
| AD-ETS4R | CTCGAGCTCGATGGATCCTCAATTAATTGACGCGGTTGACAAC |
| AD-ETS3-SP-F | CCAGATTACGCTCATATGCGGCCTGCATCAAATGCAGT |
| AD-ETS3F | CCAGATTACGCTCATATGATGGAGCTCAAGTCCCTACTCGC |
| AD-ETS3R | CTCGAGCTCGATGGATCCTTACAAGTGGCTCTTGATGAACTCG |
| AD-ETS1-SPF | CCAGATTACGCTCATATGGCCCCGGCCGGCCTCACAC |
| AD-ETS1R | CTCGAGCTCGATGGATCCTTAGATGTACGACGCTTCCAGG |
| AD-ETS2-SP-F | CCAGATTACGCTCATATGGGAAAATGCAAAGTTCCAAACCCT |
| AD-NST1R | CTCGAGCTCGATGGATCCTTAGCGAAGACGAGCATAGCCA |
| AD-ETS2-SP-F | CCAGATTACGCTCATATGGGAAAATGCAAAGTTCCAAACCCT |
| AD-ETS2R | CTCGAGCTCGATGGATCCTCAGATAGTAAAGGCAGAATAGCCCTG |
| AD-ETS6-SP-F | CCAGATTACGCTCATATGCAGCCTCCCAGAGACTCGCC |
| AD-ETS6R | CTCGAGCTCGATGGATCCTTAGATGTTGGTGCACACAGGAGG |
| AD-ETS6F | CCAGATTACGCTCATATGATGCGCTTTACTTGGATTGCTACC |
| AD-ETS7R | CTCGAGCTCGATGGATCCTCACTTGTGCCAGAACTGGACGTC |
| AD-ETS7-SP-F | CCAGATTACGCTCATATGAACTGGGTGGAAGATCACCCCGA |
| AD-ETS7F | CCAGATTACGCTCATATGATGCTTGGAAACACAATTCTTGTTGC |
| AD-ETS5-SP-F | CCAGATTACGCTCATATGGCCCCTGCCACTATTGGCC |
| AD-ETS5R | CTCGAGCTCGATGGATCCTTAGGCACCGTTGTAGGCAAG |
| AD-ETS1(102-368)-F | CCAGATTACGCTCATATGAACAAGTTGCTGGCAAACGTC |
| AD-ETS1(102-368)-R | CTCGAGCTCGATGGATCCTCATTCCAGGAGGTAGCTCACAACC |
| AD-ETS1(170-368)-F | CCAGATTACGCTCATATGATTATTGCGCGGCTGCCCGG |
| AD-ETS1(170-368)-R | CTCGAGCTCGATGGATCCTCAAAGCAGAATAGTAGGCCACATGATC |
| AD-Spz-C106F | CCAGATTACGCTCATATGGTTGGTGGCTCAGACGAGCGATTC |
| AD-Spz-R | CTCGAGCTCGATGGATCCTCACCCAGTCTTCAACGCGCAC |
| AD-Spz-F | CCAGATTACGCTCATATGAAGGAGTATGAACGTATCATCAAAGAGC |
| AD-Spz-N195-F | CCAGATTACGCTCATATGAAGGAGTATGAACGTATCATCAAA |
| AD-Spz-N195-R | CTCGAGCTCGATGGATCCTCAGCGAGAGCTCACATCCGT |
| AD-Spz-N210-F | CCAGATTACGCTCATATGAAGGAGTATGAACGTATCATCAAA |
| AD-Spz-N210-R | CTCGAGCTCGATGGATCCTCACTTCCTGATGCTCCTGCAAAG |
| AD-Toll-F | CCAGATTACGCTCATATGTCCTTCGGTCGGGATGCGTG |
| AD-Toll5B-R | CTCGAGCTCGATGGATCCTTACATATTCTTATGCAGATTGGGC |
| AD-TollVLR-R | CTCGAGCTCGATGGATCCTTACTCCAGACGCAGATCCGTCAGAT |
| AD-SPE-F | CCAGATTACGCTCATATGATTTTTGGTGGTACAAATACCACCC |
| AD-SPE-R | CTCGAGCTCGATGGATCCTCACGGCTCCAGCTTCTGTTTAATCC |
| T7 | TAATACGACTCACTATAGGGCGAGCG |
| 3AD | GTGAACTTGCGGGGTTTTTCAGTAT |
| BK-ETS6.B1-R | CGCTGCAGGTCGACGGATCCTCACGCGTCTGCAGTGTCGG |
| BK-ETS6.B2-F | GAGGAGGACCTGCATATGTGCTGCTGCTGTGATATTCG |
| BK-ETS6.B2-R | CGCTGCAGGTCGACGGATCCTCAGTTAAACCGGCGGGCAGA |
| BK-ETS6.B3-F | GAGGAGGACCTGCATATGTGCTGTTGCTGCAATCCAGG |
| BK-ETS6.B1.3-F | TGCCCGACACTGCAGACGCGTGCTGTTGCTGCAATCCAGGCAA |
| BK-ETS6.B1.3-R | TTGCCTGGATTGCAGCAACAGCACGCGTCTGCAGTGTCGGGCA |
| **Protein expression** | |
| MBP-Spz-1F | GTATTTTCAGGGCGCCATGATGAAGGAGTACGAACGCATTATT |
| MBP-Spz-1R | GTGGTGGTGGTGCTCGAGTTAGCCGGTTTTCAGGGC |
| GST-ETS1F | CAGGGGCCCCTGGGATCCATGCCTGCTGGTCTGACC |
| GST-ETS1R | TCGACCCGGGAATTCCGGTTAAATATAGCTGGCTTCCAGCAG |
| 28b-ETS1F | AGAAGGAGATATACCATGGGCATGCCTGCTGGTCTGACCCGTC |
| 28b-ETS1R | GTGGTGGTGGTGGTGGTGGTGGTGCTCGAGAATATAGCTGGCTTCCAGCAGATAAC |
| 28b-C106-His-F | AGAAGGAGATATACCATGGGCGTTGGCGGTAGCGATGAACGC |
| 28b-C106-His-R | GTGGTGGTGCTCGAGGCCGGTTTTCAGGGCACATTTG |
| 28b-MBP-Spz-F | AGAAGGAGATATACCATGGGCATGAAGGAGTACGAACGCATTATTAAGG |
| 28b-MBP-Spz-R | ATCTCAGTGGTGGTGGTGGTGGTGGTGGTGGCCGGTTTTCAGGGCACATTTGCAG |
| GST-ETS6-F | CAGGGGCCCCTGGGATCCCAGCCTCCCAGAGACTCG |
| GST-ETS6-R | TCGACCCGGGAATTCCGGTTAGATGTTGGTGCACACAGG |
| GST-ETS6.B1-F | CAGGGGCCCCTGGGATCCatgGCCCAGCCTCCCAGAGACTCG |
| GST-ETS6.B1-R | TCGACCCGGGAATTCCGGttaCGCGTCTGCAGTGTCGG |
| GST-ETS6.B2-F | CAGGGGCCCCTGGGATCCatgTGCTGCTGCTGTGATATTCG |
| GST-ETS6.B2-R | TCGACCCGGGAATTCCGGttaGTTAAACCGGCGGGCAGA |
| GST-ETS6-B3-F | CAGGGGCCCCTGGGATCCatgTGCTGTTGCTGCAATCCAGG |
| GST-ETS6-B3-R | TCGACCCGGGAATTCCGGttaTTAGATGTTGGTGCACACAGGA |
| DsSpz1C-F | AGAAGGAGATATACCATGGGCGTGGGTGGTGACCCCGACGAGAT |
| DsSpz1C-R | GTGGTGGTGCTCGAGCAGGATTTTCAGGGCGCA |
| AaSpz1C-F | AGAAGGAGATATACCATGGGCGCACCGTTTCTTTGTGAAAGCGA |
| AaSpz1C-R | GTGGTGGTGCTCGAGCGATAAAATTTTAGCACATTTACAACAGC |
| BmSpz1C-F | AGAAGGAGATATACCATGGGCGCAGGCTCATTCGAAGACTCG |
| BmSpz1C-R | GTGGTGGTGCTCGAGACCGAGTAGCGTGGCAACAC |
| **Protein secretion analysis** | |
| SUC2-ETS1-SPF | TTTAATTAAGAATTCATGCCTTCCATGTCCAAGCT |
| SUC2-ETS1-SPR | AGGGAGAACCTCGAGCGGGGCGGCGAGGCC |
| SUC2-ETS6-SPF | TTTAATTAAGAATTCATGCGCTTTACTTGGATTGCTAC |
| SUC2-ETS6-SPR | AGGGAGAACCTCGAGGGCAGCGACCGAGGAGAGGA |
| **Fly transgenesis** | |
| UAS-ETS1-F | AGATCTGCGGCCGCGGCTCGAGATGCCCAGCATGAGCAAGCTGGC |
| UAS-ETS1-R | CCTTCACAAAGATCCTCTAGATTACGTAGAATCGAGACCGAGGAGAGGGTTAGGGATAGGCTTACCGATGTAGCTGGCCTCCAGCAG |
| UAS-ETS6-F | AGATCTGCGGCCGCGGCTCGAGATGCGCTTCACCTGGATC |
| UAS-ETS6-R | CCTTCACAAAGATCCTCTAGATTACTTGTCGTCATCGTCTTTGTAGTCGATATTGGTGCACACGGGCGG |
| **Yeast three-hybrid analysis** | |
| MCS1-SPZ-F | GAATTCCCGGGGATCCGTatgAAGGAGTATGAACGTATCATCAAAGAGC |
| MCS1-SPZ-R | TGCAGGTCGACGGATCCCTCACCCAGTCTTCAACGCGCAC |
| MCS1-Spz-C106-F | GAATTCCCGGGGATCCGTGTGGGCGGAAGCGACGAGCGTTT |
| MCS1-ETS6-F | GAATTCCCGGGGATCCGTCAGCCTCCCAGAGACTCGCC |
| MCS1-ETS6-R | TGCAGGTCGACGGATCCCTTAGATGTTGGTGCACACAGGAG |
| MCS2-SPE-F | AGAAAGGTGGCGGCCGCAATTTTTGGTGGTACAAATACCACCC |
| MCS2-SPE-R | TCGGGCTAATGCGGCCGCTCACGGCTCCAGCTTCTGTTTAATCC |
| MCS2-ETS1-F | AGAAAGGTGGCGGCCGCAGCCCCGGCCGGCCTCACAC |
| MCS2-ETS1-R | TCGGGCTAATGCGGCCGCTTAGATGTACGACGCTTCCAGG |
| MCS2-ETS6-F | AGAAAGGTGGCGGCCGCACAGCCTCCCAGAGACTCGCC |
| MCS2-ETS6-R | TCGGGCTAATGCGGCCGCTTAGATGTTGGTGCACACAGGAG |
| MCS2-ETS6.B1-F | AGAAAGGTGGCGGCCGCAatgGCCCAGCCTCCCAGAGACTCG |
| MCS2-ETS6.B1-R | TCGGGCTAATGCGGCCGCTCACGCGTCTGCAGTGTCGG |
| MCS2-ETS6.B2-F | AGAAAGGTGGCGGCCGCAatgTGCTGCTGCTGTGATATTCG |
| MCS2-ETS6.B2-R | TCGGGCTAATGCGGCCGCTTAGTTAAACCGGCGGGCAGA |
| MCS2-ETS6.B3-F | AGAAAGGTGGCGGCCGCAatgTGCTGTTGCTGCAATCCAGG |
| MCS2-ETS6.B3-R | TCGGGCTAATGCGGCCGCTTAGATGTTGGTGCACACA |
| MCS2-Spz-C106-F | AGAAAGGTGGCGGCCGCAGTGGGCGGAAGCGACGAGCGTTT |
| MCS2-Spz-R | TCGGGCTAATGCGGCCGCTCACCCAGTCTTCAACGCGCAC |
| MCS2-Toll-F | AGAAAGGTGGCGGCCGCATCCTTCGGTCGGGATGCGTG |
| MCS2-TollVLR-R | TCGGGCTAATGCGGCCGCTCACTCCAGACGCAGATCCGTCAGAT |
| **RT-qPCR analysis** | |
| Drs-F | GTACTTGTTCGCCCTCTTCG |
| Drs-R | CTCCTCCTTGCACACACGAC |
| Mtk-F | GCAACTTAATCTTGGAGCGA |
| Mtk-R | CGGTCTTGGTTGGTTAGGAT |
| Daisho2-F | ATGAACTGTCTGAAGATCTGCGGC |
| Daisho2-R | GCGTATATACTGCGAGCGATCAG |
| BomS5-F | TCCTTGGTCTTTCTATGCGGT |
| BomS5-R | ACAATGACGGCAATCTCCAT |
| Daisho1-F | CTTCTCTTGGCCATGTTCGCT |
| Daisho1-R | ATGTACTGGGTGTTGTCGGTC |
| BomS1-F | CACCGTTTTTGTGCTCGGTC |
| BomS1-R | CGTGGACATTGCACACCCT |
| BaraA1-F | CCACAACACACCTACGACGG |
| BaraA1-R | CGGAAAAATTGGGACCACTG |
| BomBc2-F | GACTGGGCACTGCATCAATC |
| BomBc2-R | GCAACCGACGCAATCACTAT |
| Rp49F | GCCCAAGGGTATCGACAACA |
| Rp49R | CTTGCGCTTCTTGGAGGAGA |
| MrTubF | GGTCGCTATGAAGGAGGTTGA |
| MrTubR | TCCTGGATGGAGGTGGAGTTA |
| Spz-F | CCTTTGCAGGAGCATCAGGA |
| Spz-R | GGTCTGCTGTGTGTAGTGCT |
| UAS-ETS1-F | CGTGCAGCGTTTTAACCACA |
| UAS-ETS1-R | TCGATGGTATTCTGTGGGCG |
| UAS-ETS6-F | GGAGTCCCCGTTGTCTGG |
| UAS-ETS6-R | AGGAGTGGGCTGTTTGGC |
| ETS1-F | CGAGGTGCTTTCCATCCTGT |
| ETS1-R | GACGTTTGCCAGCAACTTGT |
| ETS6-F | GGAGTCCCCGTTGTCTGG |
| ETS6-R | AGGAGTGGGCTGTTTGGC |
